# SOX2 promotes chemoresistance, cancer stem cells properties, and epithelial–mesenchymal transition by β-catenin and Beclin1/autophagy signaling in colorectal cancer

**DOI:** 10.1038/s41419-021-03733-5

**Published:** 2021-05-05

**Authors:** Yin Zhu, Shimiao Huang, Shengyuan Chen, Jiaxuan Chen, Zhiqing Wang, Yadong Wang, Haoxuan Zheng

**Affiliations:** Guangdong Provincial Key Laboratory of Gastroenterology, Department of Gastroenterology, Nanfang Hospital, Southern Medical University, Guangzhou, China

**Keywords:** Cancer therapeutic resistance, Epithelial-mesenchymal transition

## Abstract

Sex-determining region Y-box2 (SOX2), a master regulator of embryonic and induced pluripotent stem cells, drives cancer stem cells (CSCs) properties, fuels tumor initiation, and contributes to tumor aggressiveness. Our previous study has demonstrated the oncogenic role of SOX2 in colorectal cancer (CRC). In this study, we sought to elucidate the underlying mechanisms. Cell function experiments were performed to detect chemoresistance, proliferation, stemness, migration, and invasion in vitro. Chromatin immunoprecipitation, co-immunoprecipitation, luciferase reporter assay, and immunofluorescence were performed to explore the regulation of ABCC2, β-catenin, and Beclin1 by SOX2. The carcinogenic role of SOX2-β-catenin/Beclin1-ABCC2 axis in vivo was analyzed by CRC tissues and xenograft models. Here, we reported that SOX2 sustained chemoresistance by transcriptional activation of ABCC2 expression. Suppressing either β-catenin or autophagy signaling curbed SOX2-driven chemoresistance, stemness, and epithelial–mesenchymal transition (EMT). Mechanistically, SOX2 combined with β-catenin and increased its nuclear expression and transcriptional activity. Transcriptional activation of Beclin1 expression by SOX2 consequently activating autophagy and inducing malignant phenotype. Furthermore, overexpression of β-catenin or Beclin1 facilitated ABCC2 expression. The clinical analyses showed that high expression of ABCC2 and Beclin1 were positively correlated with SOX2 and were associated with poor prognosis in CRC patients. Finally, xenograft models revealed that inhibition of SOX2 expression and autophagy restrained tumor growth and chemoresistance in vivo. Conclusively, we demonstrated a novel mechanism by which the SOX2-β-catenin/Beclin1/autophagy signaling axis regulates chemoresistance, stemness, and EMT in CRC. Our findings provide novel insights into CRC carcinogenesis and may help develop potential therapeutic candidates for CRC.

## Introduction

A large body of evidence indicates that human cancer can be categorized as a stem cell disease^1^. Cancer stem cells (CSCs) represent a minor subpopulation of the tumor bulk that exhibits self-renewal capacity, induces therapy resistance, and initiates metastasis, thereby promoting tumor progression and recurrence^2,3^. Sex-determining region Y-box2 (SOX2), a master regulator of embryonic and induced pluripotent stem cells (iPSCs), drives cancer stemness, fuels tumor initiation, and contributes to tumor aggressiveness^4^. In our recent study, we found that SOX2 promotes chemoresistance, confers CSCs properties, and promotes epithelial–mesenchymal transition (EMT) in colorectal cancer (CRC)^5^. However, the underlying mechanism of this phenomenon is not well-characterized. Elucidation of the underlying molecular mechanism may help identify novel diagnostic biomarkers and facilitate the development of effective therapeutic interventions against CRC.

It’s reported that ATP-binding cassette transporters (ABC transporters) serve as efflux pumps that extrude anti-cancer drugs from the cancer cells^6–9^. Several ABC transporters, including ABCB1, ABCC2, ABCG2, and ABCB5, have been identified in CSCs-driven chemoresistance^10–13^. Importantly, SOX2 has been implicated in the acquisition of chemoresistance by upregulating the expression of ABC transporters in glioma cells and gastric cancer cells^14,15^. However, the role of ABC transporters in SOX2-induced chemoresistance is poorly understood in CRC.

Aberrant WNT/β-catenin signaling plays an essential role in the progression of CRC^16^. SOX2 has been reported to promote EMT in laryngeal cancer and to contribute to tamoxifen resistance in breast cancer through the WNT/β-catenin pathway^17,18^. Therefore, we speculated that SOX2 may promote malignant phenotypes via the β-catenin signaling pathway in CRC.

Autophagy, which is an essential cellular mechanism for the degradation of cytoplasmic proteins and organelles and the recycling of their components, has been reported to induce resistance against chemotherapy-driven cytotoxicity via maintaining the survival of CSCs through conferring stress tolerance and limiting the damage; autophagy can regulate cell migration through selective degradation of focal adhesion proteins^19–21^. Furthermore, SOX2 has been shown to repress mTOR expression and promote cellular reprogramming through induction of autophagy in iPSCs; ectopic SOX2 expression promoted autophagosome formation and autophagic flux in head and neck squamous cell carcinoma^22,23^. However, the involvement of autophagy in SOX2-mediated malignant phenotypes in CRC is not well-characterized. Besides, autophagy has been shown to participate in the degradation of β-catenin or to activate the WNT/β-catenin pathway^24,25^. The cross-talk between autophagy and β-catenin in SOX2-induced malignant phenotypes in CRC deserves further exploration.

In this study, we aimed to investigate the mechanism of how SOX2 induces chemoresistance, CSCs properties, and EMT in CRC. Our findings may provide new insights into CRC carcinogenesis and help identify autophagy-related therapeutic targets for CRC.

## Materials and methods

### Cell culture and reagents

Human CRC SW480 and SW620 cell lines were obtained from the American Type Culture Collection (ATCC) and authenticated according to the recommendations of ATCC and grown in RPMI 1640 (Gibco, C11875500BT) containing 10% fetal bovine serum (Gibco, 10099-141, FBS) in a humidified 5% CO_2_ atmosphere at 37°C. Human embryonal kidney 293 cells (HEK293T) were preserved at the Nanfang Hospital, Southern Medical University (Guangzhou, China) and cultivated in a DMEM (Gibco, C11885500CP) medium supplemented with 10% FBS. SN38 (Sigma-Aldrich, H0165), 5-Fu (Selleck.cn, S1209), and oxaliplatin (Selleck.cn, S1224) were used at a concentration of 4 µM, 15 µg/mL, and 60 µM, respectively. Rapamycin (Sigma-Aldrich, 37094) (50 nM) was used to activate autophagy, while 3-methyladenine (Sigma-Aldrich, M9281, 3-MA) (10 mM) was used to block autophagy.

### Cell line transfection

The SOX2, ABCC2, β-catenin, or Beclin1 construction were generated by sub-cloning PCR-amplified full-length human SOX2 (NM_003106), ABCC2 (NM_000392.5), β-catenin (NM_001098210.2) or Beclin1 (NM_001313998.2) cDNA into plasmid Ubi-MCS-3FLAG-SV40-EGFP-IRES-puromycin, Ubi-MCS-3FLAG-SV40-Cherry-IRES-neomycin, Ubi-MCS-3FLAG-SV40-Cherry-IRES-neomycin or pLOV-EF1a-mcherry-P2A-Puro-CMV, respectively. To knock down endogenous SOX2 or ABCC2, 2 short hairpin RNA (shRNA) (Supplementary Table S1) were cloned into the hU6-MCS-Ubiquitin-EGFP-IRES-puromycin or hU6-MCS-Ubiquitin-mCherry-IRES-Neomycin vectors to generate hU6-MCS-Ubiquitin-EGFP-IRES-SOX2-RNAi(s) or hU6-MCS-Ubiquitin-mCherry-IRES-ABCC2-RNAi(s). Endogenous β-catenin or Beclin1 were knocked down by designed small interfering RNA (siRNA) (Supplementary Table S2). We co-transfected RNAi-1 and RNAi-2 of SOX2 or ABCC2 into SW620 cells to achieve maximum knockdown efficacy. Similarly, we co-transfected siRNA-1 and siRNA-2 of β-catenin and Beclin1 into SW620 cells to achieve maximum knockdown efficacy (Supplementary Fig. S2). The co-transfected SW620 cells were used for subsequent experiments.

### Quantitative real-time PCR (qRT-PCR), western blot, drug sensitivity assay, cell proliferation assay, sphere formation assay, and transwell invasion assay

qRT-PCR, western blot, cell proliferation assay, and transwell invasion assay were performed, as previously described^26^. Drug sensitivity assay and sphere formation assay were performed as described before^5^. The primers for qRT-PCR are listed in the Supplementary Tables S3 section and the antibodies for western blot are as following: GAPDH (1:1000; Cell Signaling Technology, 3683S), SOX2 (1:1000; Cell Signaling Technology, 23064S), ABCC2 (1:1000; Abcam, ab3373), β-catenin (1:1000; Abcam, ab22656), CD133 (1:1000; Abcam, ab19898), Snail (1:1000; Cell Signaling Technology, 3879S), Vimentin (1:1000; Abcam, ab92547), N-cadherin (1:1000; Cell Signaling Technology, 13116S), E-cadherin (1:1000; Cell Signaling Technology, 3195S), Histone (1:2000; Cell Signaling Technology, 4499S), c-Myc (1:1000; Cell Signaling Technology, 9402S), cyclin D1 (1:1000; Abcam, ab16663), Axin2 (1:1000; Abcam, ab32197), LC3B (1:1000; Cell Signaling Technology, 43566S), P62 (1:1000; Cell Signaling Technology, 16177S), ULK1 (1:1000; Abcam, ab203207), Beclin1 (1:1000; Abcam, ab114071), ATG5 (1:1000; Cell Signaling Technology, 2630S), ATG7 (1:1000; Cell Signaling Technology, 2631S), ATG10 (1:1000; Abcam, ab124711), ATG12 (1:1000; Abcam, ab109491), anti-rabbit IgG (1:5000; Cell Signaling Technology, 7074S), anti-mouse IgG (1:5000; Cell Signaling Technology, 7076S). Experiments were conducted in triplicate.

### Wound-healing assay

CRC cells (2 × 10^5^) were seeded in each well of a 6-well plate. Upon attainment of 90–100% confluence, the cell monolayer was scratched in a straight line using a sterile pipette tip, followed by careful washing to remove the detached cells. Subsequently, the cells were cultured at 37 °C with 5% CO_2_. Photographs of the scratch wound in different samples were recorded at 0 and 48 h.

### Chromatin immunoprecipitation (ChIP)

ChIP assay was performed using the EZ-ChIP kit (Millipore, 17-371), and the purified DNA fragments were subjected to PCR amplification set, as previously described^27^. The primers for PCR are listed in the Supplementary Tables S4 section.

### Nuclear cytoplasmic fractionation assay

Nuclear and cytoplasmic proteins of SW480 and SW620 cells were extracted using the Nuclear and Cytoplasmic Protein Extraction Kit (Beyotime, P0027), according to the manufacturer’s protocol. For Western blot analyses, GAPDH and Histone were used as cytoplasmic and nuclear control markers, respectively.

### Co-immunoprecipitation (co-IP)

2 μg of the anti-SOX2 (Cell Signaling Technology, 23064S) or anti-β-catenin antibody (Cell Signaling Technology, 2677S) was added to 1 mg of the protein lysates and rotated overnight at 4 °C. An equal amount of protein lysates incubated with a non-specific IgG antibody was used as the negative control. An equal amount of protein lysate was used as the positive control. The immune complexes were precipitated by protein A/G beads (Santa Cruz Biotechnology, sc-2003) overnight at 4 °C. Beads with extracted proteins were washed 3 times with cold PBS, followed by CelLytic M lysis buffer 1 time (Sigma-Aldrich, C2978). The bound proteins were eluted by boiling at 95 °C for 5 min with 20 μL of SDS protein buffer and then processed for Western blot.

### Immunofluorescence and confocal microscopy

The cells grown on coverslips in a 24-well plate were washed twice with cold PBS, and then fixed with 4% paraformaldehyde for 15 min at room temperature (RT). Subsequently, the cells were permeabilized with 0.1% Triton X-100 for 5 min on ice, and stained with following antibodies: SOX2 (1:400; Cell Signaling Technology, 23064S), β-catenin (1:200; Cell Signaling Technology, 2677S), anti-mouse IgG/PE (1:100; Bioss, bs-0296G-PE) and anti-rabbit IgG/FITC (1:100; Bioss, bs-0295G- FITC). Nuclei were stained with 4′6-diamidino-2-phenylindole (DAPI) dye. Images were obtained with laser scanning confocal microscopy (Olympus, FV1000, Japan). The cells were transiently transfected with mRFP-GFP-LC3B adenovirus (Hanbio Co. Ltd., HB-AP2100001) for 6 h; subsequently, the number of LC3B puncta was analyzed by confocal microscopy (Olympus, FV1000, Japan).

### Luciferase reporter assay

The ABCC2 promoter reporter (Guangzhou Kidan BioTechnology Co. Ltd.), Beclin1 promoter reporter (Guangzhou Kidan BioTechnology Co. Ltd), or TOP/FOP Flash reporter containing TCF/LEF1 binding sites (GeneCopoeia) was introduced into cultured cells using Lipofectamine 3000 (Invitrogen, L3000-008), according to the manufacturer’s instructions. After 48 h, cells were lysed, and luciferase activity was assessed with Dual Luciferase Reporter Assay Kit (Beyotime, RG027), as per the manufacturer’s instructions.

### Transmission electron microscopy (TEM)

CRC cells were cultured in 10-cm-dishes for 24 h and fixed in 2.5% glutaraldehyde in 100 mM phosphate buffer for 8 h at 25 °C, and re-fixed in 1% osmic acid for 3 h at 4 °C. Subsequently, the cells were embedded in Spurr’s resin (Ted-Pella, 18300), sectioned, doubly stained with uranyl acetate and lead citrate, and analyzed using a transmission electron microscope (JEOL, JEM1230, Japan).

### Tissue microarray

Tissue microarray with 90 pairs of CRC tissues (Shanghai Outdo Biotech Co. Ltd.) were used to assess the expression of SOX2, ABCC2, and Beclin1 using immunohistochemistry (IHC). The intensity of staining of malignant cells was scored as follows to analyze the levels of protein expression: + (no staining), ++ (weak staining), +++ (moderate staining), and ++++ (strong staining). An intensity score greater than + + was classified as high expression, whereas a score of less than or equal to ++ was considered as low expression, as described elsewhere^28^.

### Animal experiments

SW620 cells stably transfected with SOX2 short hairpin RNA (shRNA) or negative shRNA (5 × 10^6^ cells in 0.1 mL PBS) were subcutaneously injected into the right dorsal flanks of 4-week-old female Balb/c nude mice (*n* = 3/group), respectively. Twenty-two days later, mice bearing tumors were administered intraperitoneal injection of PBS, Chloroquine (Sigma-Aldrich, C6628, CQ, 20 mg/kg), irinotecan (Sigma-Aldrich, I1406, 20 mg/kg), or combination of irinotecan and CQ every 3 days. Tumor size was measured with a digital caliper every 3 days. Tumor volume was calculated from digital caliper measurements of tumor dimensions in mm using the formula for a prolate ellipsoid: (*L* × *W*^2^)/2, where *L* is the longer of the 2 measurements. At the endpoint, tumors were harvested and weighed. The excised tissues were either fixed in 10% neutral-buffered formalin or snap frozen in liquid nitrogen. Tumor sections from paraffin-embedded blocks were used for histologic examination. All animal experiments were conducted as per the protocol approved by the Animal Care and Use Committee of the Southern Medical University.

### Statistical analysis

Statistical analysis was performed using SPSS 21.0. Between-group differences were assessed using one-way ANOVA or independent sample *t*-test. The difference in the expression of each molecule in ranked data was calculated using the Chi-squared test. Survival curves were plotted based on the follow-up data using the Kaplan–Meier method, and cumulative survival rates in different groups were compared using the log-rank test. The correlation coefficient was calculated using the Spearman method. *P* values ≤ 0.05 were considered indicative of statistical significance.

## Results

### SOX2 transcriptionally activates ABCC2 expression and induces chemoresistance in CRC

**Fig. 1:**
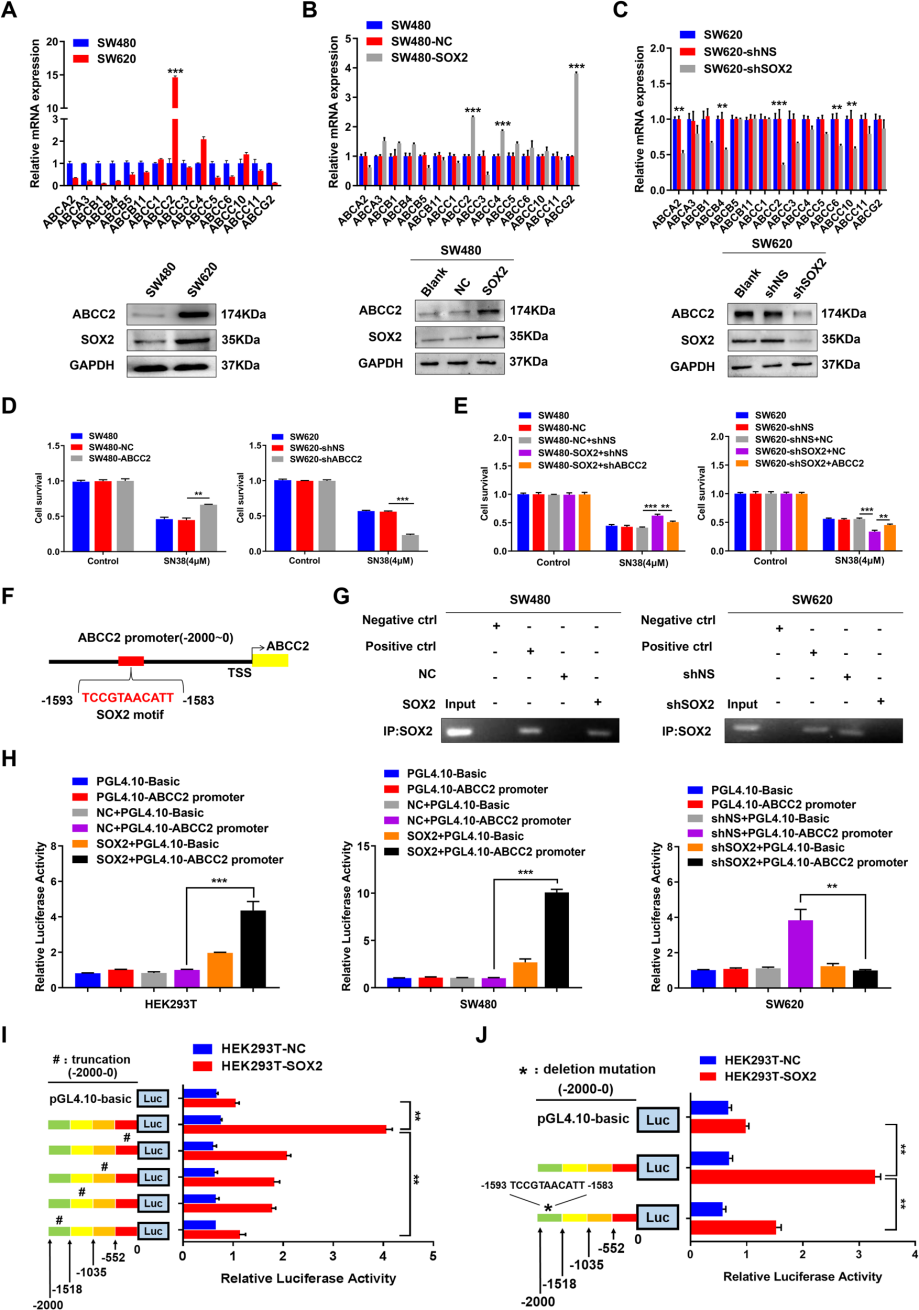
SOX2 transcriptionally activates ABCC2 expression and induces chemoresistance in CRC. **A**–**C** mRNA and protein level of different ABC transporters were examined in SW480 and SW620 cells (**A**), SW480 cells transfected with SOX2 clone (**B**) as well as in SW620 cells transfected with SOX2 shRNA (**C**). **D** Chemoresistance was analyzed by drug sensitivity assay with SN38 in ABCC2-overexpressing SW480 cells and ABCC2-silencing SW620 cells. **E** ABCC2 overexpression or knockdown was conducted in SW480 and SW620 cells transfected with SOX2 clone or SOX2 shRNA, then chemoresistance was analyzed by drug sensitivity assay with SN38. **F** Schematic map of potential SOX2-binding site in the promoter of ABCC2 was shown according to the JASPAR database. **G** ChIP assay was used to confirm the interaction between SOX2 and ABCC2 promoter in SW480 and SW620 cells transfected with SOX2 clone or SOX2 shRNA. IgG antibody was used as a negative control, RNA polymerase II antibody was used as a positive control. **H** ABCC2 promoter-driven luciferase activity was assessed in HEK293T, SW480, and SW620 cells transfected with SOX2 clone or SOX2 shRNA. **I**, **J** Luciferase activity of ABCC2 promoter was examined in HEK293T cells transfected with truncated ABCC2 promoter (**I**) or deletion mutant of ABCC2 promoter (**J**). Experiments were conducted in triplicate. Data are shown as mean ± SEM. *P < 0.05, **P < 0.01, and ***P < 0.001.

Gene set enrichment analysis (GSEA) indicated that there was a positive correlation between SOX2 high expression and ABC transporters signatures (TCGA, *n* = 465, Supplementary Fig. S1A). qRT-PCR and western blot revealed that ABCC2 was markedly elevated in SW620 cells than that in SW480 cells (Fig. 1A). Then, SW480 cells with a low level of SOX2 were transfected with the SOX2 clone vector, while SW620 cells with a high level of SOX2 were transfected with SOX2 shRNA (Supplementary Fig. S2A). Overexpression of SOX2 led to a dramatic increase in ABCC2 expression (Fig. 1B), while knockdown of SOX2 led to decreased ABCC2 expression (Fig. 1C). Moreover, ABCC2 overexpression in SW480 cells increased the resistance to SN38 (the active metabolite of irinotecan and the substrate of ABCC2), whereas ABCC2 knockdown in SW620 cells did the opposite (Fig. 1D and Supplementary Fig. S2B). To further elucidate the contribution of ABCC2 to SOX2-driven chemoresistance, we reversed ABCC2 expression in SW480 and SW620 cells stably transfected with SOX2 clone vector or SOX2 shRNA (Supplementary Fig. S2C). The results showed that reversing ABCC2 expression attenuated SOX2-induced chemoresistance in CRC cells (Fig. 1E).

It’s well known that SOX2 is a transcription factor, we sought to identify potential SOX2-binding sites in the ABCC2 promoter by JASPAR database (http://jaspar.genereg.net/). The results showed that there was a potential SOX2-binding site in the promoter of ABCC2 (−1593~−1583, upstream of transcriptional starting site, TSS, Fig. 1F). ChIP assay and luciferase reporter assay with full-length ABCC2 promoter (−2000~0) further verified the binding of SOX2 on ABCC2 promoter (Fig. 1G, H). Next, we constructed four partial truncations of the ABCC2 promoter region (0~−552, −552~−1035, −1035~−1518, −1518~−2000). Luciferase reporter assay showed that transcriptional activity significantly decreased in the fourth truncated region of ABCC2 promoter (−1518~−2000, Fig. 1I), where was exactly the predicted binding site located (−1593~−1583). Furthermore, deletion mutation of this sequence (−1593~−1583) markedly restrained SOX2-induced luciferase activity of ABCC2 promoter (Fig. 1J).

Collectively, these data suggested that SOX2 promoted chemoresistance partly through transcriptional activation of ABCC2 expression in CRC cells.

### β-catenin contributes to SOX2-mediated chemoresistance, CSCs properties, and EMT in CRC

**Fig. 2:**
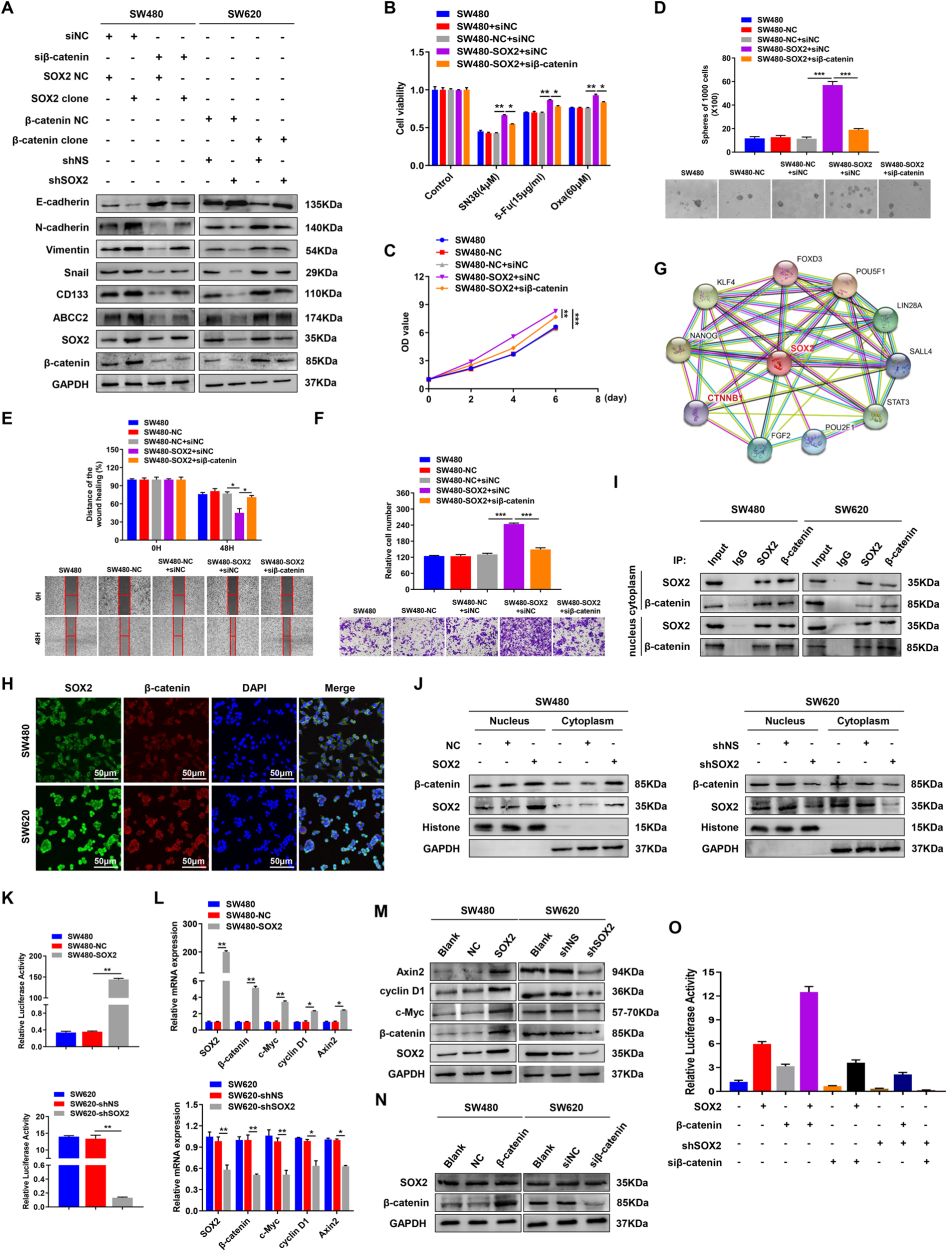
β-catenin contributes to SOX2-mediated chemoresistance, CSCs properties, and EMT in CRC. **A** Overexpression or knockdown of β-catenin was conducted in SW480 and SW620 cells transfected with SOX2 clone or SOX2 shRNA, then immunoblot was performed with indicated antibodies. **B**–**F** The ability of chemoresistance (**B**), proliferation (**C**), stemness (**D**), migration (**E**), and invasion (**F**) in SW480 cells co-transfected with SOX2 clone and β-catenin siRNA was assessed by drug sensitivity assay, cell viability assay, tumor sphere formation assay, wound healing assay and transwell invasion assay. **G** β-catenin is a potential binding partner of SOX2 predicted by the STRING database. **H** Immunofluorescence was performed to analyze the co-localization of SOX2 and β-catenin in SW480 and SW620 cells. **I** co-IP assay was used to analyze the interaction between SOX2 and β-catenin in both cytoplasm and a nuclear fraction of SW480 and SW620 cells. **J** Western blot detected the expression of SOX2 and β-catenin in the nucleus and cytoplasm of SOX2-overexpressing SW480 cells and SOX2-silencing SW620 cells. **K** TOP/FOP Flash reporter assay was used to detected β-catenin transcriptional activity in SW480 and SW620 cells transfected with SOX2 clone or SOX2 shRNA. **L**, **M** Total expression of β-catenin and its target genes (c-Myc, cyclin D1, and Axin2) was detected by qRT-PCR (**L**) and western blot (**M**) in SOX2-overexpressing SW480 cells and SOX2-silencing SW620 cells. **N** Effect of β-catenin overexpression or knockdown on SOX2 expression was detected by western blot in SW480 and SW620 cells. **O** Effect of β-catenin overexpression or knockdown on SOX2-mediated ABCC2 promoter transactivation was assessed in HEK293T cells by luciferase reporter assay. Experiments were conducted in triplicate. Data are shown as mean ± SEM. *P < 0.05, **P < 0.01, and ***P < 0.001.

The results of GSEA analysis suggested a strong link between SOX2 and β-catenin (TCGA, *n* = 465, Supplementary Fig. S1B). To further validate the promoting effect of β-catenin on chemoresistance, CSCs properties, and EMT in CRC, SW480 cells with low β-catenin expression were transfected with β-catenin overexpression plasmid, while SW620 cells with high β-catenin expression were transfected with β-catenin small interfering RNA (siRNA) (Supplementary Fig. S2D). Ectopic β-catenin overexpression dramatically increased chemoresistance, proliferation, stemness, and metastasis in SW480 cells; downregulation of β-catenin in SW620 cells showed the opposite effect (Supplementary Fig. S3A–F). To determine the role of β-catenin in SOX2-mediated malignant phenotype in CRC, we reversed β-catenin expression in SW480 and SW620 cells stably transfected with SOX2 clone vector or SOX2 shRNA (Supplementary Fig. S2E). The results showed that β-catenin downregulation suppressed the expression of ABCC2, CD133, and mesenchymal markers, but increased the expression of epithelial markers in SOX2-overexpressing SW480 cells; however, β-catenin overexpression in SOX2-silencing SW620 cells had the opposite effect (Fig. 2A). Consistently, knockdown of β-catenin partly abrogated the induction of chemoresistance, CSCs properties, and EMT in SOX2-overexpressing SW480 cells (Fig. 2B–F), whereas upregulation of β-catenin partly eliminated the inhibition of chemoresistance, CSCs properties, and EMT in SOX2-silencing SW620 cells (Supplementary Fig. S3G–K).

Collectively, these findings indicate that the oncogenic role of β-catenin was involved in SOX2-mediated chemoresistance, CSCs properties, and EMT in CRC.

### SOX2 combines with β-catenin and upregulates β-catenin nuclear expression and transcriptional activity

As β-catenin can mediate SOX2-induced malignant phenotypes, we sought to determine the mechanism of SOX2 regulating β-catenin. Noticeably, β-catenin is a potential binding partner of SOX2 predicted by the STRING database (https://string-db.org/) (Fig. 2G). Immunofluorescence assay revealed the co-localization of SOX2 and β-catenin in the cytoplasm and nucleus of SW480 or SW620 cells (Fig. 2H). Moreover, endogenous SOX2 and β-catenin could be co-immunoprecipitated with each other in the cytoplasm and nucleus fraction of the above two type CRC cells (Fig. 2I). These results suggested that SOX2 combined with β-catenin in CRC cells. Upregulation of β-catenin nuclear expression which binds to TCF/LEF1 transcription factors can lead to activation of target genes, which are essential for CRC tumorigenesis and progression^29^. We next explored the influence of SOX2 on β-catenin nuclear expression and its transcriptional activity. The results revealed that SOX2 overexpression in SW480 cells increased nuclear and cytoplasmic β-catenin expression, β-catenin transcriptional activity, and β-catenin target genes (c-Myc, cyclin D1, and Axin2) expression; in contrast, SOX2 knockdown in SW620 cells had the opposite effect (Fig. 2J–M). Upregulation or downregulation of β-catenin did not alter the expression of SOX2 in SW480 or SW620 cells (Fig. 2N). Moreover, co-transfection of SOX2 and β-catenin had a synergistic effect on ABCC2 promoter activity; knockdown of either β-catenin or SOX2 partly reduced ABCC2 promoter-driven luciferase activity and diminished the synergy between SOX2 and β-catenin; suggesting a functional linkage of SOX2 with β-catenin in the transcriptional regulation of ABCC2 in CRC (Fig. 2O).

These findings demonstrated that SOX2 combined with β-catenin increased β-catenin nuclear expression and transcriptional activity in CRC. Moreover, as a transcriptional partner, β-catenin acted in synergy with SOX2 in the transcriptional regulation of ABCC2 in CRC.

### SOX2 promotes chemoresistance, CSCs properties, and EMT partly via autophagy in CRC

**Fig. 3:**
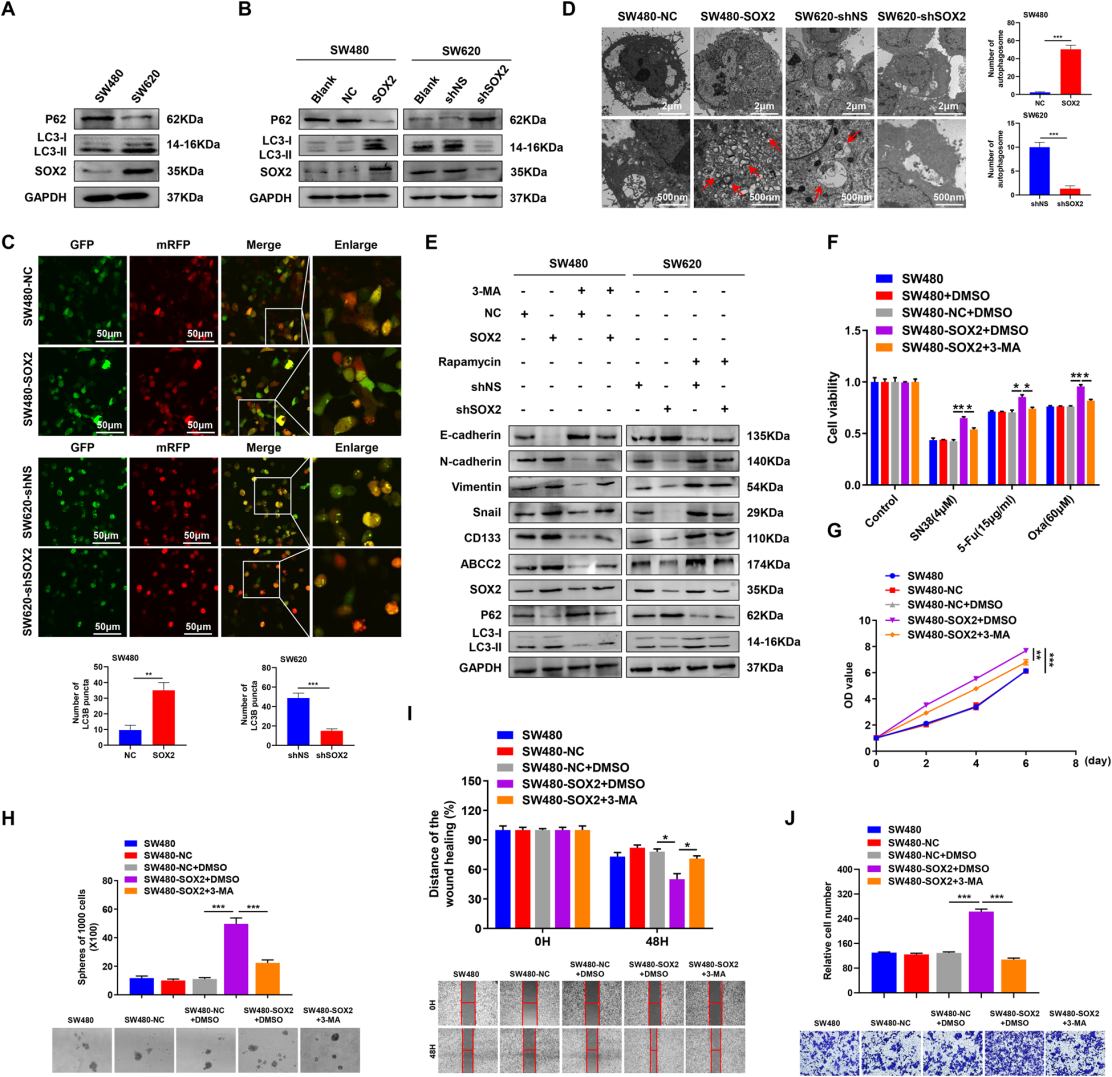
Autophagy is involved in SOX2-induced chemoresistance, CSCs properties, and EMT in CRC. **A**, **B** Western blot analysis of LC3II/LC3I and P62 expression in SW480 and SW620 cells (**A**) as well as in SW480 and SW620 transfected with SOX2 clone or SOX2 shRNA (**B**). **C** SOX2-overexpressing SW480 cells or SOX2-deficient SW620 cells were transiently transfected with mRFP-GFP-LC3B adenovirus for 6 h, then the number of LC3B puncta was analyzed by confocal microscopy. **D** Transmission electron microscopy was utilized to assess the number of autophagic vesicles in SW480 and SW620 cells transfected with SOX2 clone or SOX2 shRNA. Red arrows indicated autophagic vesicles. **E** SW480 or SW620 cells transfected with SOX2 clone or SOX2 shRNA were treated with 3-MA (10 mM) or Rapamycin (50 nM) for 24 h, then western blot was performed with indicated antibodies. **F**–**J** The ability of chemoresistance (**F**), proliferation (**G**), stemness (**H**), migration (**I**), and invasion (**J**) in SOX2-overexpressing SW480 cells treated with 3-MA was examined by drug sensitivity assay, cell viability assay, tumor sphere formation assay, wound healing assay, and transwell invasion assay. Experiments were conducted in triplicate. Data are shown as mean ± SEM. *P < 0.05, **P < 0.01, and ***P < 0.001.

GSEA plot indicated that “GO_REGULATION_OF_AUTOPHAGY” and “GO_ POSITIVE_REGULATION_OF_AUTOPHAGY” gene signatures enriched in the SOX2 high expression group, suggesting the interaction between SOX2 and autophagy (TCGA, *n* = 465, Supplementary Fig. S1C). Western blot showed that SW620 cells exhibited increased autophagy marker MAP1LC3B/LC3B (microtubule-associated protein 1 light chain 3 beta) and decreased autophagy substrate SQSTM1/P62 expression as compared to SW480 cells (Fig. 3A), indicating enhanced autophagic flux in SW620 cells. Moreover, western blot and fluorescent imaging analyses revealed that overexpression of SOX2 in SW480 cells significantly increased the autophagic flux and LC3B puncta formation, while downregulation of SOX2 in SW620 did the opposite (Fig. 3B, C). Consistently, transmission electron microscopy showed a dramatic increase in the number of autophagic vesicles in SOX2-overexpressing SW480 cells, but a decrease in SOX2-silenced SW620 cells (Fig. 3D). These results suggested that SOX2 may enhance autophagy in CRC cells. Importantly, treatment of SW480 cells with 50 nM rapamycin (autophagy activator) increased chemoresistance, proliferation, stemness, migration, and invasion of SW480 cells; treatment of SW620 cells with 10 mM 3-MA (autophagy inhibitor) had the opposite effects (Supplementary Fig. S4A–F). Additionally, to address the role of autophagy in SOX2-induced malignant phenotypes in CRC, we inhibited autophagy with 3-MA in SOX2-overexpressing SW480 cells and activated autophagy with rapamycin in SOX2-silencing SW620 cells. The results showed that SOX2-driven malignant phenotype was partially diminished by autophagy inhibition, but partially restored by autophagy activation (Fig. 3E–J and Supplementary Fig. S4G–K). These findings demonstrated the involvement of autophagy in SOX2-driven malignant phenotypes in CRC.

### SOX2 activates autophagy by transcriptional promoting Beclin1 expression

**Fig. 4:**
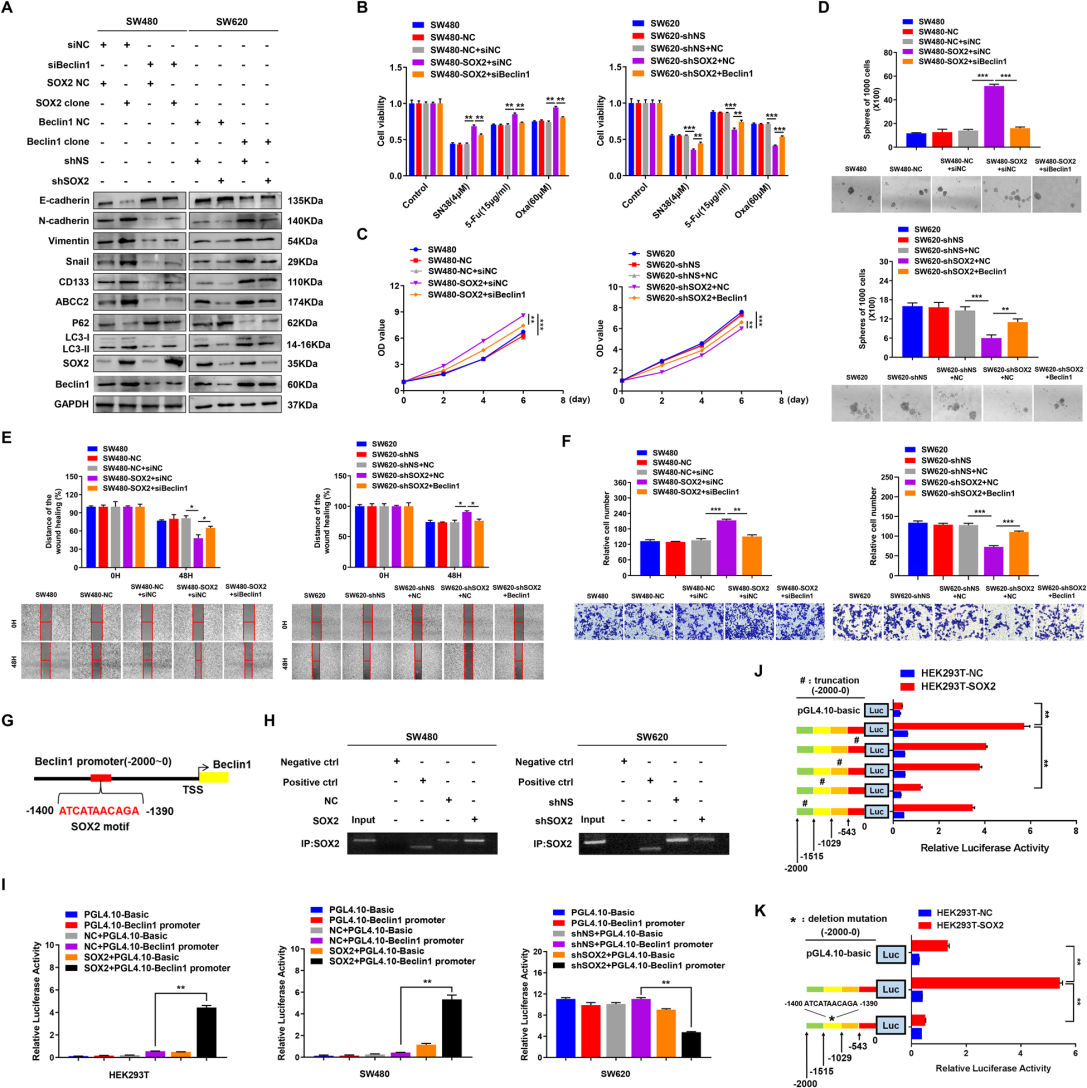
Beclin1 is responsible for SOX2-induced autophagy activation, chemoresistance, CSCs properties, and EMT in CRC. **A** Overexpression or knockdown of Beclin1 was conducted in SW480 or SW620 cells transfected with SOX2 clone or SOX2 shRNA, then western blot was performed with indicated antibodies. **B**–**F** The ability of chemoresistance (**B**), proliferation (**C**), stemness (**D**) migration (**E**), and invasion (**F**) was examined in SW480 cells co-transfected with SOX2 clone and Beclin1 siRNA or in SW620 cells co-transfected with SOX2 shRNA and Beclin1 clone by drug sensitivity assay, cell viability assay, tumor sphere formation assay, wound healing assay, and transwell invasion assay, respectively. **G** Schematic map of potential SOX2-binding site in the promoter of Beclin1 was shown according to the JASPAR database. **H** ChIP assay analysis of the interaction between SOX2 and Beclin1 promoter in SW480 and SW620 cells transfected with SOX2 clone or SOX2 shRNA. IgG antibody was used as a negative control, RNA polymerase II antibody was used as a positive control. **I** Beclin1 promoter-driven luciferase activity was assessed in HEK293T, SW480, and SW620 cells transfected with SOX2 clone or SOX2 shRNA. **J**, **K** Luciferase activity of Beclin1 promoter was examined in HEK293T cells transfected with truncated Beclin1 promoter (**J**) or deletion mutant of Beclin1 promoter (**K**). Experiments were conducted in triplicate. Data are shown as mean ± SEM. *P < 0.05, **P < 0.01, and ***P < 0.001.

It’s reported that autophagy is a complex metabolic process regulated by multiple autophagy-related genes. By analyzing the expression of autophagy-related genes, we found that BECN1 (homolog of yeast Atg6 in humans) was marked upregulation in SW620 cells than that in SW480 cells (Supplementary Fig. S5A). Moreover, SOX2 overexpression promoted Beclin1 expression in SW480 cells, whereas SOX2 knockdown inhibited Beclin1 expression in SW620 cells (Supplementary Fig. S5B). To further investigate the role of Beclin1 in CRC malignant phenotypes, we generated Beclin1-overexpressing SW480 cells and Beclin1-deficient SW620 cells (Supplementary Fig. S2F). Similar to the effect of rapamycin or 3-MA treatment, overexpression of Beclin1 markedly increased the autophagy flux, chemoresistance, stemness and EMT, whereas knockdown of Beclin1 did the opposite (Supplementary Fig. S5C–H). These findings indicated the oncogenic role of Beclin1 in CRC. Most importantly, downregulation of Beclin1 suppressed autophagy flux and malignant phenotypes in SW480 cells with SOX2 overexpression, while upregulation of Beclin1 reversed the inhibition of autophagy and malignant phenotypes in SOX2-silencing SW620 cells (Supplementary Fig. S2G and Fig. 4A–F). These findings suggested that Beclin1 involve in SOX2-induced autophagy activation and aggressive phenotype in CRC.

Next, we sought to investigate the mechanism of how SOX2 regulates Beclin1. JASPAR database analyses revealed that SOX2 could bind on Beclin1 promoter (−1400~−1390, upstream of TSS) (Fig. 4G). ChIP assay and luciferase reporter assay with the full-length promoter (−2000~0) of Beclin1 further confirmed the binding of SOX2 on Beclin1 promoter (Fig. 4H, I). Besides, luciferase activity assay with partial truncated Beclin1 promoter (0~−543, −543~−1029, −1029~−1515, −1515~−2000) and corresponding predicted SOX2-binding sequence (−1400~−1390) deletion mutation demonstrated that SOX2 can bind to Beclin1 promoter (Fig. 4J, K).

**Fig. 5:**
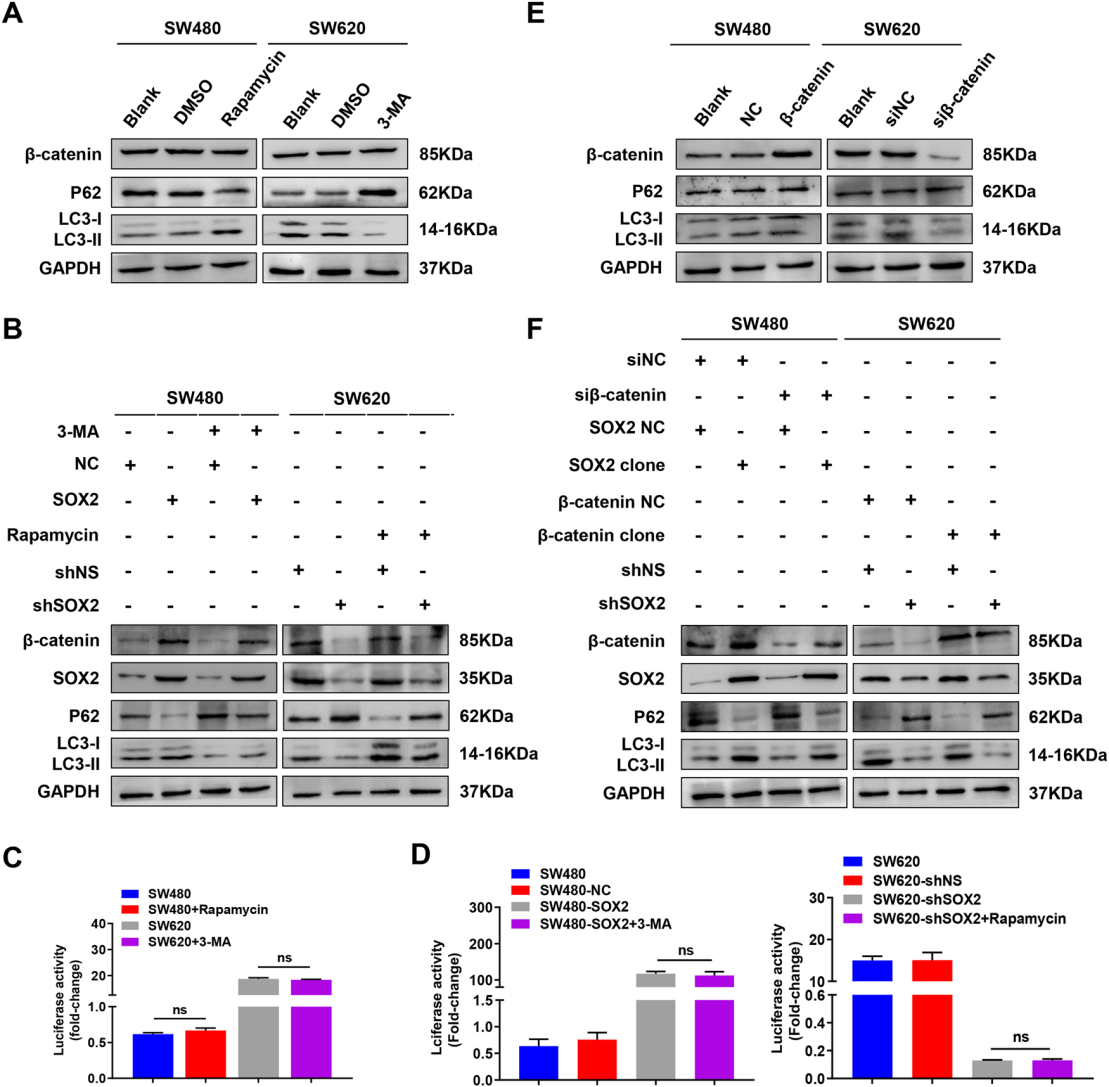
Autophagy and β-catenin signaling cannot interfere with each other in SOX2-induced malignant phenotype in CRC. **A**, **B** Activating or inhibiting autophagy by Rapamycin (50 nM) or 3-MA (10 mM) treatment for 24 h was performed in SW480 or SW620 cells (**A**) as well as in SW480 or SW620 cells transfected with SOX2 clone or SOX2 shRNA (**B**), then immunoblot was performed with indicated antibodies. **C**, **D** TOP/FOP Flash reporter assay was used to analyze β-catenin transcriptional activity in SW480 or SW620 cells with autophagy activation or inhibition (**C**) as well as in SOX2-overexpressing SW480 cells or SOX2-silencing SW620 cells with autophagy inhibition or activation (**D**). **E**, **F** Overexpressing or silencing β-catenin was conducted in SW480 or SW620 cells (**E**), as well as in SW480 or SW620 cells transfected with SOX2 clone or SOX2 shRNA (**F**), then immunoblot was performed with indicated antibodies. Experiments were conducted in triplicate. Data are shown as mean ± SEM. ns non-significant.

Recent studies have reported that autophagy bilaterally regulates β-catenin. Thus, it’s necessary to explore the relationship between autophagy and β-catenin in CRC. However, we found no distinct effect of autophagy on β-catenin expression and transcriptional activity in SW480 and SW620 cells, as well as in SW480 and SW620 cells transfected with SOX2 clone or SOX2 shRNA (Fig. 5A–D). Besides, upregulation or downregulation of β-catenin also did not alter the level of autophagy in the above CRC cells (Fig. 5E, F).

In general, these results revealed that SOX2 activated autophagy by transcriptional promoting Beclin1 expression. Moreover, autophagy and β-catenin signaling pathways were not found to interfere with each other in SOX2-induced malignant phenotypes in CRC.

### Expression of SOX2, ABCC2, and Beclin1 in clinical samples

**Fig. 6:**
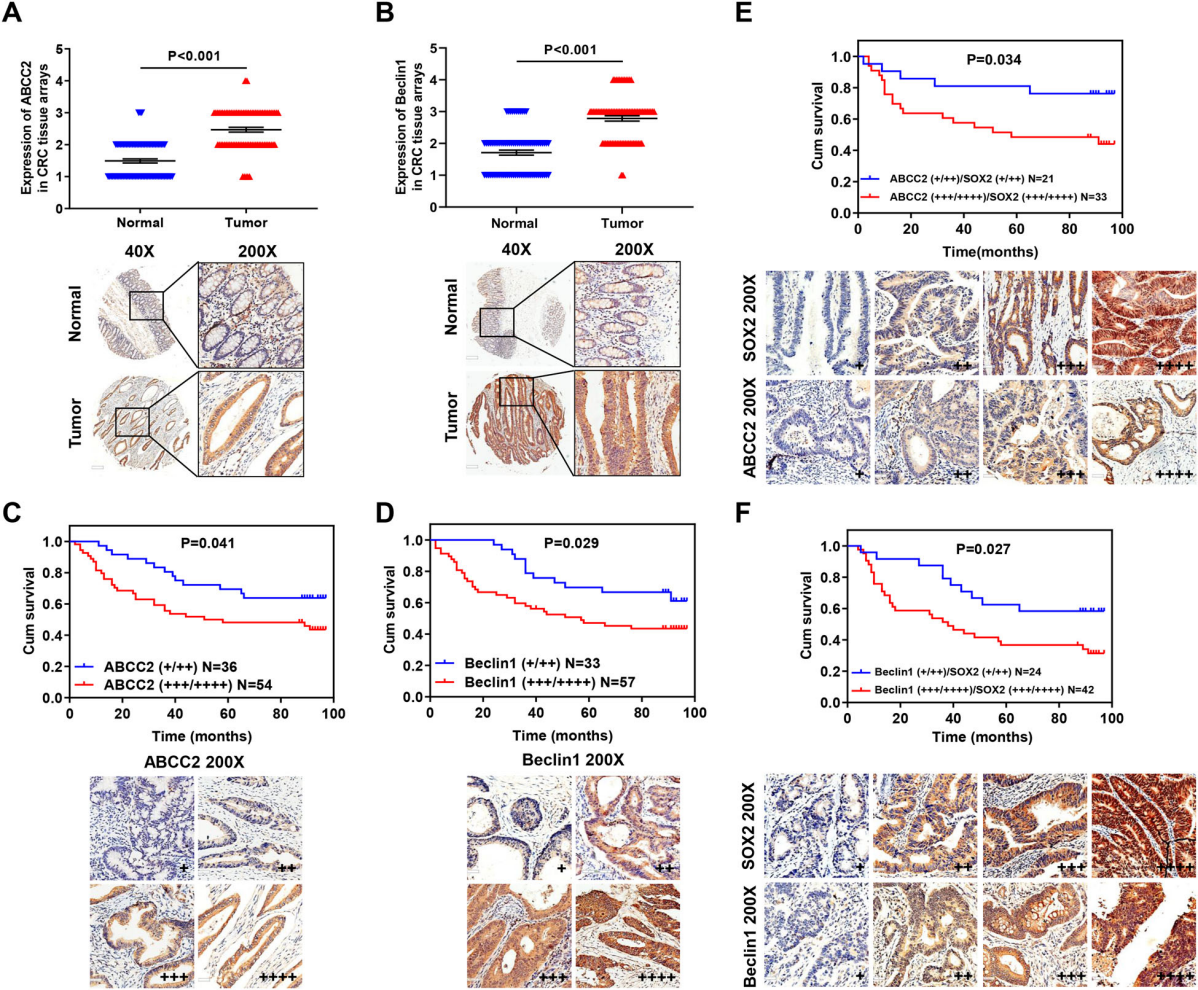
The expression of SOX2, ABCC2, and Beclin1 in clinical samples. **A**, **B** The protein expression of ABCC2 (**A**) and Beclin1 (**B**) were examined by IHC in a microarray with 90 pairs of CRC tissues. **C**–**F** Survival curves were generated with Kaplan–Meier method according to the expression of ABCC2 (**C**), Beclin1 (**D**), both ABCC2 and SOX2 (**E**) as well as both Beclin1 and SOX2 (**F**). Log-rank analysis was used to test for significance.

Microarray with 90 pairs of CRC tissue analyses showed that ABCC2 and Beclin1 expression were significantly upregulated in CRC tissues (Fig. 6A, B). In addition, ABCC2 overexpression in clinical samples was associated with lymph metastasis and TNM stage (AJCC) (Supplementary Table S5, *P* < 0.05), and Beclin1 overexpression in clinical samples was associated with TNM stage (AJCC) (Supplementary Table S6, *P* < 0.05). The survival duration of patients with high ABCC2 or Beclin1 expression was significantly shorter than that of patients with low expression (Fig. 6C, D). Moreover, patients with low expression of both SOX2 and ABCC2 had a better prognosis than those with high expression (Fig. 6E). This was also true for patients with the same expression of both SOX2 and Beclin1 (Fig. 6F). Furthermore, ABCC2 or Beclin1 expression showed a positive correlation with SOX2 expression in CRC samples (Tables 1 and 2).

**Table 1:** Correlation between SOX2 and ABCC2 expression in CRC patients.

		ABCC2 scoring		Correlation coefficient (*R*)
		Low expression (+/++) (*n* = 38)	High expression (+++/++++) (*n* = 52)	
SOX2	Low expression (+/++) (*n* = 40)	21	19	R = 0.228
Scoring	High expression (+++/++++) (*n* = 40)	17	33	P = 0.031

**Table 2:** Correlation between SOX2 and Beclin1 expression in CRC patients.

		Beclin1		Correlation coefficient (*R*)
		Low expression (+/++) (*n* = 36)	High expression (+++/++++) (*n* = 54)	
SOX2	Low expression (+/++) (*n* = 36)	24	12	R = 0.42
Scoring	High expression (+++/++++) (*n* = 54)	12	42	P = 0.00002

Overall, the expression of ABCC2 and Beclin1 were upregulated and showed a positive correlation with SOX2 expression in CRC patients, predicting poor prognosis.

### SOX2-induced autophagy promotes tumor growth and chemoresistance in vivo

**Fig. 7:**
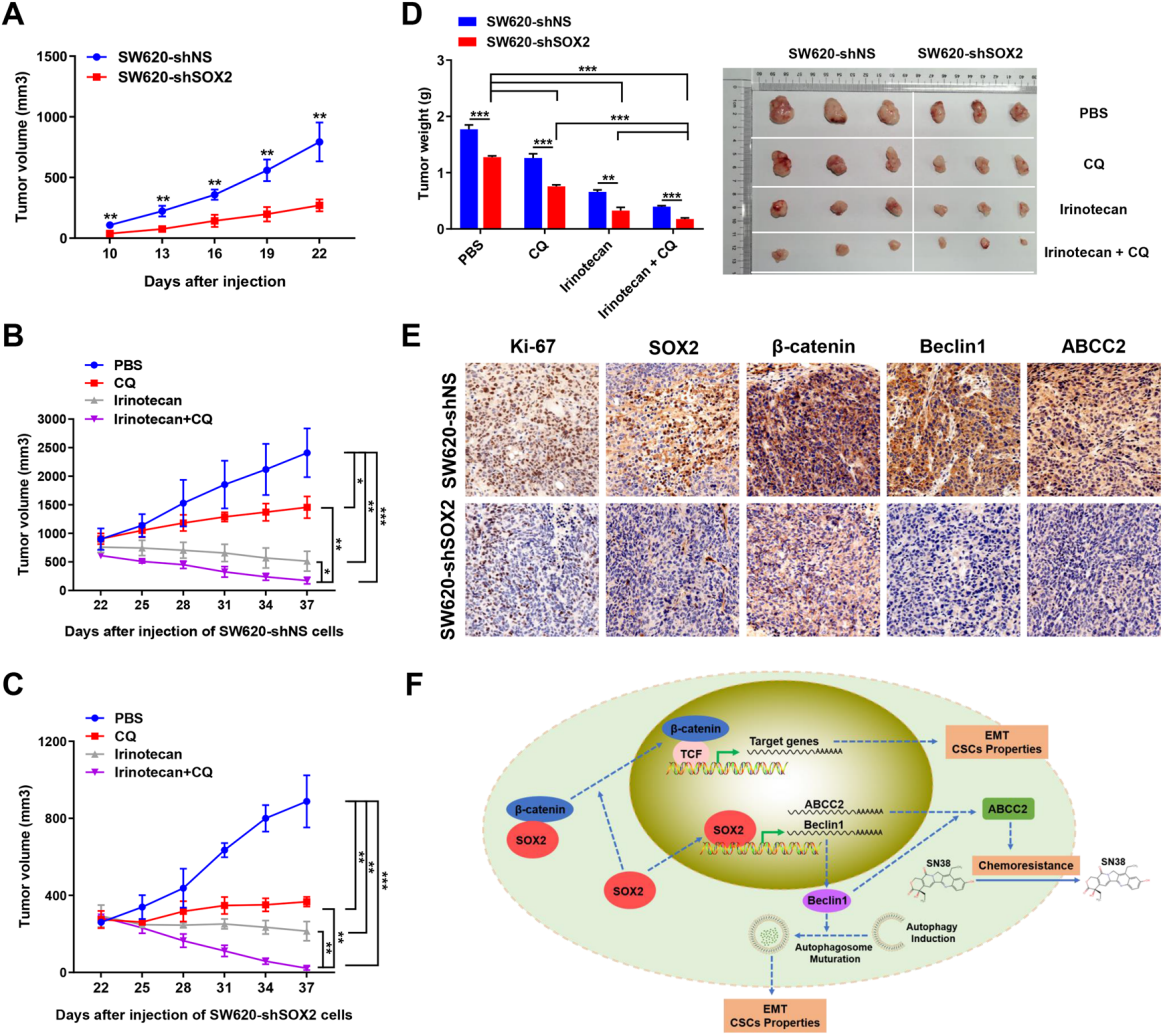
Knockdown of SOX2-restrained tumor growth and chemoresistance in vivo. **A**–**C** Growth curves of xenografts with SOX2-silencing SW620 cells and control SW620 cells were plotted based on tumor volume. **D** Weight of xenografts was measured at the endpoint. **E** IHC analysis of SOX2, β-catenin, Beclin1, and ABCC2 expression in xenografts. **F** Schematic representation of the proposed mechanism for SOX2-induced chemoresistance, CSCs properties, and EMT in CRC. Data are shown as mean ± SEM. *P < 0.05, **P < 0.01, ***P < 0.001.

According to the in vitro findings, we sought to assess the effect of SOX2-β-catenin/Beclin1/autophagy signaling-ABCC2 axis in mouse xenograft models. On the 22nd day after injection, mice injected with SW620 control cells produced larger tumors (~800 mm^3^) than mice injected with SOX2-silenced SW620 cells (~300 mm^3^) (Fig. 7A), indicating that SOX2 knockdown markedly decreased tumor growth. Autophagy inhibitor CQ has been used in many recent clinical trials. Therefore, we used CQ to investigate the effect of autophagy inhibition on tumor growth. On the 22nd day after injection, tumor-bearing mice were treated with PBS, CQ, irinotecan, or a combination of irinotecan and CQ. The results showed that mice treated with irinotecan in combination with CQ had much smaller tumor than mice treated with CQ or irinotecan alone (Fig. 7B–D). These results demonstrated that blockage of autophagy may increase tumor chemosensitivity to irinotecan in vivo. Besides, the chemotherapy effect of irinotecan combined with CQ treatment on the tumor in the SW620 SOX2 KD group was better than that in the SW620 control group, suggesting that SOX2 knockdown may increase tumor chemosensitivity in vivo (Supplementary Table S7). IHC staining demonstrated that SOX2 downregulation was accompanied by reduced β-catenin, Beclin1, and ABCC2 expression in xenografts (Fig. 7E).

In summary, these results suggested that SOX2-β-catenin/Beclin1/autophagy signaling axis plays an essential role in CRC growth and chemoresistance in vivo.

## Discussion

The persistence of CSCs promotes ABC transporters expression and induces EMT, resulting in chemoresistance, metastasis, and poor prognosis in CRC^30,31^. Our previous study has demonstrated that SOX2 promotes chemoresistance, CSCs properties, and EMT in CRC^5^. In this study, we sought to elucidate the underlying mechanism by which SOX2 regulates malignant phenotypes in CRC. Our findings demonstrated that SOX2-β-catenin/Beclin1/autophagy signaling axis is crucial for chemoresistance, CSCs properties, and EMT in CRC, which may help develop potential therapeutic candidates for CRC treatment.

SOX2 is credited with catalyzing pro-survival and anti-apoptotic signaling in a diverse range of cancers, which ultimately lead to therapeutic resistance and clinical relapse^32^. Resistance to anti-tumor therapy is associated with SOX2-mediated activation of ABC transporters, which are able to efflux drugs across the cell membrane by utilizing ATP^8,14^. However, the role of ABC transporters in SOX2-induced chemoresistance in CRC is poorly understood. Our findings indicated that ABCC2 is a direct functional target of SOX2 in CRC. SOX2 promotes chemoresistance by transcriptionally upregulating ABCC2 expression, as well as via β-catenin and autophagy signaling, respectively.

Aberrant β-catenin signaling plays an essential role in the progression of CRC^16^. Consistently, we found that β-catenin serves as an oncogenic factor in CRC. Moreover, β-catenin knockdown partly abrogated the activation of chemoresistance, CSCs properties, and EMT in SOX2-overexpressing SW480 cells; in contrast, β-catenin upregulation partly eliminates the inhibition of chemoresistance, CSCs properties, and EMT in SOX2-silencing SW620 cells. These results indicated the involvement of β-catenin in SOX2-mediated malignant phenotypes in CRC. Subsequently, we investigated the mechanism of how SOX2 regulates β-catenin in CRC. A previous study indicated that β-catenin is a transcription partner for SOX2, and that the combination of SOX2 with β-catenin promotes the transcriptional activity of each other in breast cancer^33^. Besides, SOX2 has been shown to bind to the promoter of β-catenin to promote the expression of β-catenin^34^. Similarly, we found that SOX2 combines with β-catenin and increases β-catenin total expression, nuclear accumulation, and transcriptional activity in CRC. Additionally, as a transcriptional partner, β-catenin acts in synergy with SOX2 in the transcriptional regulation of ABCC2 in CRC.

Autophagy is a highly regulated process aimed at the degradation and recycling of cellular components^35^. Emerging evidence indicates that autophagy acts as a “double-edged sword” in the context of cancer. On the one hand, autophagy suppresses tumorigenesis by clearing the damaged proteins and organelles, and perhaps by maintaining energy homeostasis through intracellular recycling; this ultimately decreases the accumulation of deleterious mutations that drives tumorigenesis^36–38^. On the other hand, autophagy confers tumor cells with superior stress tolerance that limits damage, maintains viability, sustains dormancy, and facilitates recovery^39,40^. Herein, we showed that autophagy activation promotes chemoresistance, proliferation, stemness, and metastasis in CRC cells. Most importantly, SOX2-induced chemoresistance, CSCs properties, and EMT were partially diminished by inhibition of autophagy, which suggested the involvement of autophagy in SOX2-induced malignant phenotypes in CRC. Subsequently, we investigated the mechanism of how SOX2 regulates autophagy. Autophagy is a complex metabolic process regulated by multiple autophagy-related genes. BECN1 is the first identified mammalian autophagy-mediated gene involved in the formation of autophagosomes^41^. In this study, SOX2 was found to transcriptionally induce Beclin1 expression and activate autophagy in CRC. Recent studies have reported that autophagy participates in the degradation of β-catenin or activation of the WNT/β-catenin pathway^24,25^. The inconsistent cross-talk between autophagy and β-catenin may be attributable to carcinoma heterogeneity. Herein, we showed that autophagy and β-catenin signaling pathways do not interfere with each other in SOX2-mediated malignant phenotypes. In other words, SOX2 can promote chemoresistance, CSCs properties, and EMT in CRC via β-catenin and autophagy signaling, respectively.

According to our in vitro findings, we further assessed the IHC expression of SOX2, ABCC2, and Beclin1 in a microarray with 90 pairs of clinical CRC tissues. Elevated SOX2 expression has been reported in many cancers, such as osteosarcoma, lung and esophageal squamous cell carcinoma^42,43^. Consistently, we found upregulation of SOX2 in CRC tissues compared to the adjacent normal tissues^5^. ABCC2 is expressed at a high level in tamoxifen-resistant breast cancer, and ABCC2 upregulation suggests a poor prognosis in pancreatic cancer^44,45^. Similarly, we found elevated expression of ABCC2 in CRC tissues. However, different studies have shown conflicting roles of Beclin1 in tumors. Decreased expression of Beclin1 is frequently found in human breast, ovarian, and prostate cancers^46,47^, while 95% of colon cancer tissues have high expression of Beclin1^48,49^. This discrepancy is largely attributable to the differences in the cancer cells and tumor models used in various studies. In this study, CRC tissues analysis showed significantly increased expression of Beclin1. Furthermore, ABCC2 or Beclin1 is positively correlated with SOX2 expression and associated with TNM stage (AJCC), predicting poor prognosis in CRC patients. Besides, we verified the effect of SOX2-β-catenin/Beclin1/autophagy signaling-ABCC2 axis in tumor growth and chemoresistance in vivo via mouse models. CQ is a well-established autophagy inhibitor. Our results demonstrated that the combination of irinotecan and CQ therapy increased tumor chemosensitivity to irinotecan. Moreover, the chemotherapy effect of irinotecan combined with CQ treatment on the tumor in SW620 SOX2 KD group is better than that in the SW620 control group, suggesting that SOX2 knockdown may increase tumor chemosensitivity in vivo.

In summary, we demonstrate a novel mechanism whereby the SOX2-β-catenin/Beclin1/autophagy signaling axis regulates chemoresistance, CSC properties, and EMT in CRC (Fig. 7F). Hopefully, these findings may contribute to the development of new therapeutic candidates for CRC and provide potentially useful experimental evidence for autophagy-based cancer therapy.

## Supplementary information

Supplementary Figures and Figure Legends

Supplementary Tables

## References

[1] Dalerba P (2007). Phenotypic characterization of human colorectal cancer stem cells. Proc. Natl Acad. Sci. USA.

[2] Singh SK (2004). Identification of human brain tumour initiating cells. Nature.

[3] López de Andrés, J., Griñán-Lisón, C., Jiménez, G. & Marchal, J. A. Cancer stem cell secretome in the tumor microenvironment: a key point for an effective personalized cancer treatment. *J. Hematol. Oncol.* **13**, 136 (2020). 10.1186/s13045-020-00966-3 PMC7559894 33059744

[4] Favaro R (2014). Sox2 is required to maintain cancer stem cells in a mouse model of high-grade oligodendroglioma. Cancer Res..

[5] Chen J, Chen S, Zhuo L, Zhu Y, Zheng H (2020). Regulation of cancer stem cell properties, angiogenesis, and vasculogenic mimicry by miR-450a-5p/SOX2 axis in colorectal cancer. Cell Death Dis..

[6] Moitra K, Lou H, Dean M (2011). Multidrug efflux pumps and cancer stem cells: insights into multidrug resistance and therapeutic development. Clin. Pharmacol. Therap..

[7] Lai Q (2020). CTCF promotes colorectal cancer cell proliferation and chemotherapy resistance to 5-FU via the P53-Hedgehog axis. Aging.

[8] Kathawala RJ, Gupta P, Ashby CR, Chen ZS (2015). The modulation of ABC transporter-mediated multidrug resistance in cancer: a review of the past decade. Drug Resist. Updat..

[9] Huang L (2013). Induction of acquired drug resistance in endothelial cells and its involvement in anticancer therapy. J. Hematol. Oncol..

[10] Nobili S (2020). Role of ATP-binding cassette transporters in cancer initiation and progression. Semin. Cancer Biol..

[11] Sugano T (2015). Inhibition of ABCB1 overcomes cancer stem cell-like properties and acquired resistance to MET inhibitors in non-small cell lung cancer. Mol. Cancer Therap..

[12] Warrier S, Pavanram P, Raina D, Arvind M (2012). Study of chemoresistant CD133+ cancer stem cells from human glioblastoma cell line U138MG using multiple assays. Cell Biol. Int..

[13] Wilson BJ (2014). ABCB5 maintains melanoma-initiating cells through a proinflammatory cytokine signaling circuit. Cancer Res..

[14] Jeon HM (2011). ID4 imparts chemoresistance and cancer stemness to glioma cells by derepressing miR-9*-mediated suppression of SOX2. Cancer Res..

[15] Tian T, Zhang Y, Wang S, Zhou J, Xu S (2012). Sox2 enhances the tumorigenicity and chemoresistance of cancer stem-like cells derived from gastric cancer. J. Biomed. Res..

[16] Bian, J., Dannappel, M., Wan, C. & Firestein, R. Transcriptional regulation of Wnt/β-catenin pathway in colorectal cancer. *Cells* **9**, 2125 (2020). 10.3390/cells9092125 PMC7564852 32961708

[17] Yang N, Hui L, Wang Y, Yang H, Jiang X (2014). Overexpression of SOX2 promotes migration, invasion, and epithelial-mesenchymal transition through the Wnt/β-catenin pathway in laryngeal cancer Hep-2 cells. Tumour Biol..

[18] Piva M (2014). Sox2 promotes tamoxifen resistance in breast cancer cells. EMBO Mol. Med..

[19] Yang MC (2015). Blockade of autophagy reduces pancreatic cancer stem cell activity and potentiates the tumoricidal effect of gemcitabine. Mol. Cancer.

[20] Pagotto A (2017). Autophagy inhibition reduces chemoresistance and tumorigenic potential of human ovarian cancer stem cells. Cell Death Dis..

[21] Sharifi MN (2016). Autophagy promotes focal adhesion disassembly and cell motility of metastatic tumor cells through the direct interaction of Paxillin with LC3. Cell Rep..

[22] Wang S (2013). Transient activation of autophagy via Sox2-mediated suppression of mTOR is an important early step in reprogramming to pluripotency. ell Stem Cell.

[23] Tan YS (2018). Mitigating SOX2-potentiated Immune Escape of Head and Neck Squamous Cell Carcinoma with a STING-inducing Nanosatellite Vaccine. Clin. Cancer Res..

[24] Wu H (2019). TRAF6 inhibits colorectal cancer metastasis through regulating selective autophagic CTNNB1/β-catenin degradation and is targeted for GSK3B/GSK3β-mediated phosphorylation and degradation. Autophagy.

[25] Fan Q (2018). Autophagy promotes metastasis and glycolysis by upregulating MCT1 expression and Wnt/β-catenin signaling pathway activation in hepatocellular carcinoma cells. J. Exp. Clin. Cancer Res..

[26] Zheng HX (2013). Fas signaling promotes motility and metastasis through epithelial-mesenchymal transition in gastrointestinal cancer. Oncogene.

[27] Zheng H (2014). miR-23a inhibits E-cadherin expression and is regulated by AP-1 and NFAT4 complex during Fas-induced EMT in gastrointestinal cancer. Carcinogenesis.

[28] Wang B (2017). LASP2 suppresses colorectal cancer progression through JNK/p38 MAPK pathway meditated epithelial-mesenchymal transition. Cell Commun. Signal..

[29] Schuijers J, Mokry M, Hatzis P, Cuppen E, Clevers H (2014). Wnt-induced transcriptional activation is exclusively mediated by TCF/LEF. EMBO J..

[30] Dalerba P, Cho RW, Clarke MF (2007). Cancer stem cells: models and concepts. Annu. Rev. Med..

[31] Mani SA (2008). The epithelial-mesenchymal transition generates cells with properties of stem cells. Cell.

[32] Saigusa S (2009). Correlation of CD133, OCT4, and SOX2 in rectal cancer and their association with distant recurrence after chemoradiotherapy. Ann. Surg. Oncol..

[33] Chen Y (2008). The molecular mechanism governing the oncogenic potential of SOX2 in breast cancer. J. Biol. Chem..

[34] Li X (2013). SOX2 promotes tumor metastasis by stimulating epithelial-to-mesenchymal transition via regulation of WNT/β-catenin signal network. Cancer Lett..

[35] Klionsky DJ, Emr SD (2000). Autophagy as a regulated pathway of cellular degradation. Science.

[36] White E (2012). Deconvoluting the context-dependent role for autophagy in cancer. Nat. Rev. Cancer.

[37] Karantza-Wadsworth V (2007). Autophagy mitigates metabolic stress and genome damage in mammary tumorigenesis. Genes Dev..

[38] Mathew R (2007). Autophagy suppresses tumor progression by limiting chromosomal instability. Genes Dev..

[39] Degenhardt K (2006). Autophagy promotes tumor cell survival and restricts necrosis, inflammation, and tumorigenesis. Cancer Cell.

[40] Guo JY (2011). Activated Ras requires autophagy to maintain oxidative metabolism and tumorigenesis. Genes Dev..

[41] Funderburk SF, Wang QJ, Yue Z (2010). The Beclin 1-VPS34 complex-at the crossroads of autophagy and beyond. Trends Cell Biol..

[42] Basu-Roy U (2012). Sox2 maintains self renewal of tumor-initiating cells in osteosarcomas. Oncogene.

[43] Bass AJ (2009). SOX2 is an amplified lineage-survival oncogene in lung and esophageal squamous cell carcinomas. Nat. Genet..

[44] Choi HK, Yang JW, Roh SH, Han CY, Kang KW (2007). Induction of multidrug resistance associated protein 2 in tamoxifen-resistant breast cancer cells. Endocr.Relat. Cancer.

[45] Tanaka M, Okazaki T, Suzuki H, Abbruzzese JL, Li D (2011). Association of multi-drug resistance gene polymorphisms with pancreatic cancer outcome. Cancer.

[46] Saito H (1993). Detailed deletion mapping of chromosome 17q in ovarian and breast cancers: 2-cM region on 17q21.3 often and commonly deleted in tumors. Cancer Res..

[47] Gao X (1995). Loss of heterozygosity of the BRCA1 and other loci on chromosome 17q in human prostate cancer. Cancer Res..

[48] Ahn CH (2007). Expression of beclin-1, an autophagy-related protein, in gastric and colorectal cancers. APMIS.

[49] Zhang MY (2014). Beclin 1 expression is closely linked to colorectal carcinogenesis and distant metastasis of colorectal carcinoma. Int J. Mol. Sci..

