# Supplementary material for: SOX2 promotes chemoresistance, cancer stem cells properties, and epithelial–mesenchymal transition by β-catenin and Beclin1/autophagy signaling in colorectal cancer
Source: Cell Death Dis. 2021 May 5;12(5):449. doi: 10.1038/s41419-021-03733-5 (PMC8100126; doi:10.1038/s41419-021-03733-5)
Supplement: Supplementary file 1 — Supplementary Figures and Figure Legends [file 41419_2021_3733_MOESM1_ESM.docx]

**Supplementary Figures and Figure Legends**

**
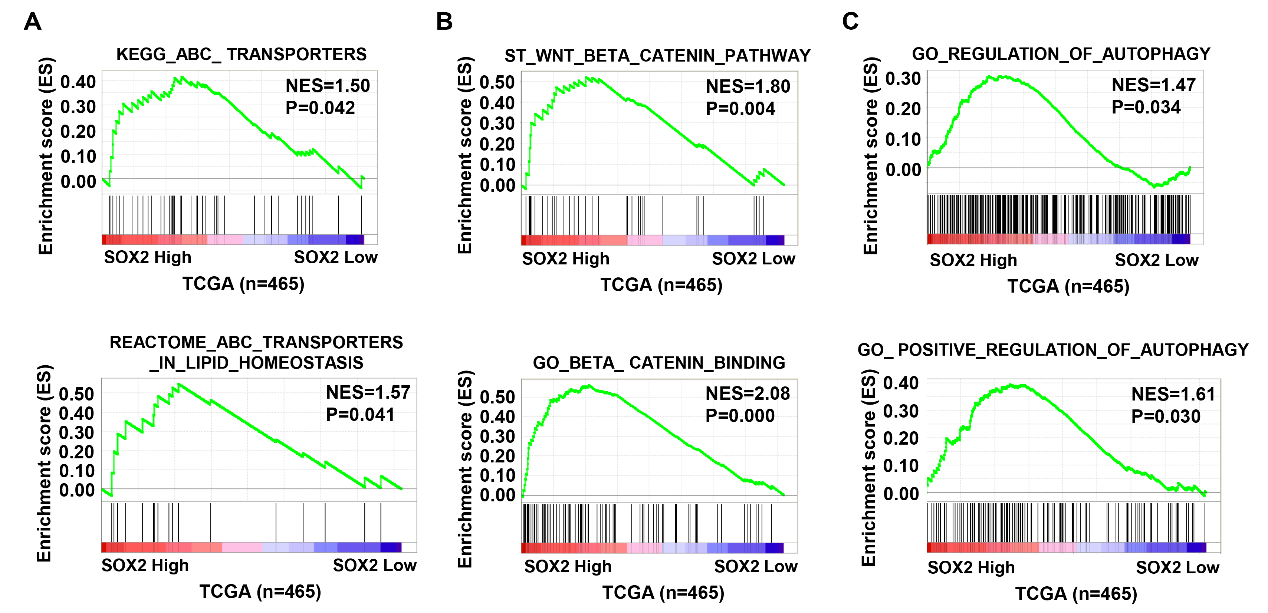
**

**Supplementary Figure S1. GSEA analysis of SOX2-regulated gene set signatures.**

**A-C,** GSEA indicated that high expression of SOX2 was positively correlated with “KEGG_ABC_ TRANSPORTERS” and “REACTOME_ABC_TRANSPORTERS_IN_LIPID_HOMEO STASIS” (A), “ST_WNT_BETA_CATENIN_PATHWAY” and “GO_BETA_CATENIN_ BINDING” (B), “GO_REGULATION_OF_AUTOPHAGY” and “GO_POSITIVE_REGULATION_OF_ AUTOPHAGY” (C) gene set signatures in CRC patients gene expression profiles (TCGA, n = 465).

**
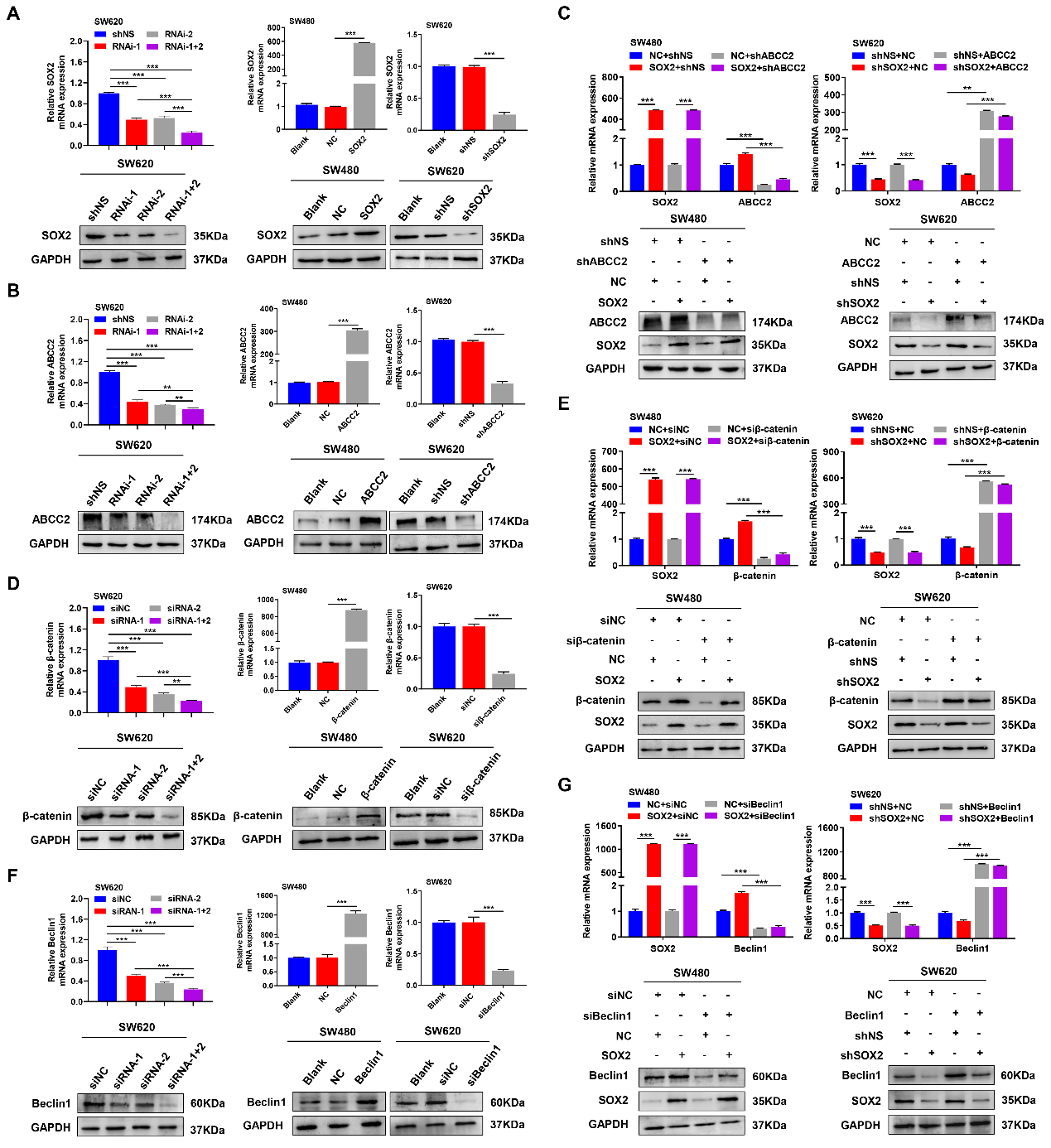
**

**Supplementary Figure S2. The efficacy of genes transfection.**

**A and B,** Efficacy of SOX2 (A) and ABCC2 (B) transfection in SW480 and SW620 cells was assessed by qRT-PCR and western blot. We co-transfected RNAi-1 and RNAi-2 of SOX2 or ABCC2 into SW620 cells to achieve maximum knockdown efficacy and the co-transfected SW620 cells were used for subsequent experiments. **C,** Overexpression or knockdown of ABCC2 was conducted in SW480 or SW620 cells transfected with SOX2 clone or SOX2 shRNA, then genes transfection efficacy was assessed by qRT-PCR and western blot. **D,** The efficacy of β-catenin transfection in SW480 and SW620 cells was assessed by qRT-PCR and western blot. We co-transfected siRNA-1 and siRNA-2 of β-catenin into SW620 cells to achieve maximum knockdown efficacy and the co-transfected SW620 cells were used for subsequent experiments. **E,** Overexpression or knockdown of β-catenin was conducted in SW480 or SW620 cells transfected with SOX2 clone or SOX2 shRNA, then genes transfection efficacy was assessed by qRT-PCR and western blot. **F,** The efficacy of Beclin1 transfection in SW480 and SW620 cells was assessed by qRT-PCR and western blot. We co-transfected siRNA-1 and siRNA-2 of Beclin1 into SW620 cells to achieve maximum knockdown efficacy and the co-transfected SW620 cells were used for subsequent experiments. **G,** Overexpression or knockdown of Beclin1 was conducted in SW480 or SW620 cells transfected with SOX2 clone or SOX2 shRNA, then genes transfection efficacy was assessed by qRT-PCR and western blot. Experiments were performed in triplicate. Data are shown as mean ± SEM. * P<0.05，**P<0.01, and ***P<0.001.


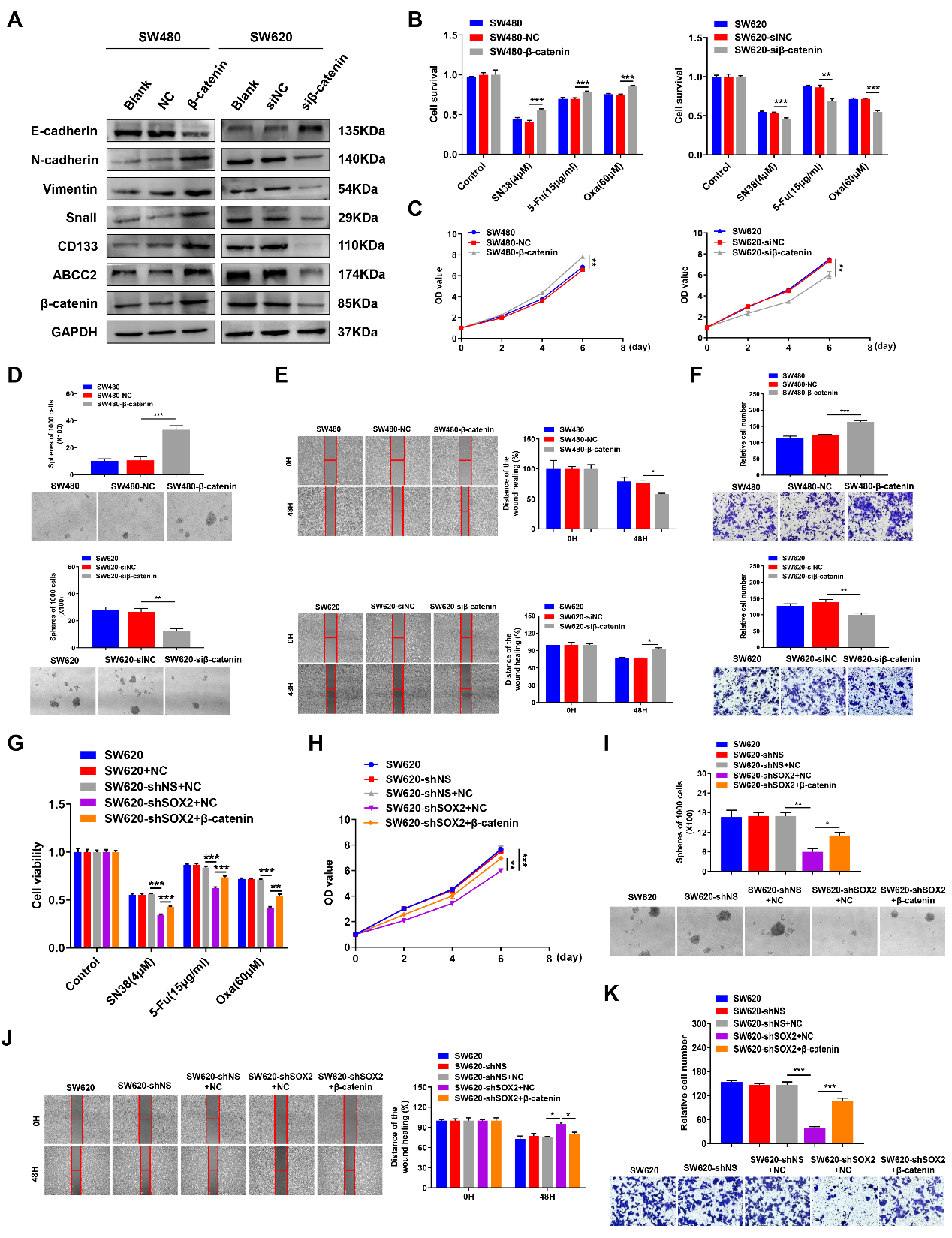


**Supplementary Figure S3.** **SOX2 promotes chemoresistance,** **CSCs properties and** **EMT partly via β-catenin in** **CRC.**

**A,** Overexpressing or silencing β-catenin was conducted in SW480 or SW620 cells, then western blot was performed with indicated antibodies. **B-F,** The ability of chemoresistance (B), proliferation (C), stemness (D) migration (E) and invasion (F) was assessed in SW480 or SW620 cells transfected with β-catenin clone or β-catenin siRNA by drug sensitivity assay, cell viability assay, tumor sphere formation assay, wound healing assay and transwell invasion assay, respectively. **G-K,** Upregulating β-catenin was conducted in SW620 cells with stable SOX2 knockdown, then the ability of chemoresistance (G), proliferation (H), stemness (I) migration (J) and invasion (K) was assessed by drug sensitivity assay, cell viability assay, tumor sphere formation assay, wound healing assay and transwell invasion assay, respectively. Experiments were conducted in triplicate. Data are shown as mean ± SEM. *P<0.05, **P<0.01, ***P<0.001.


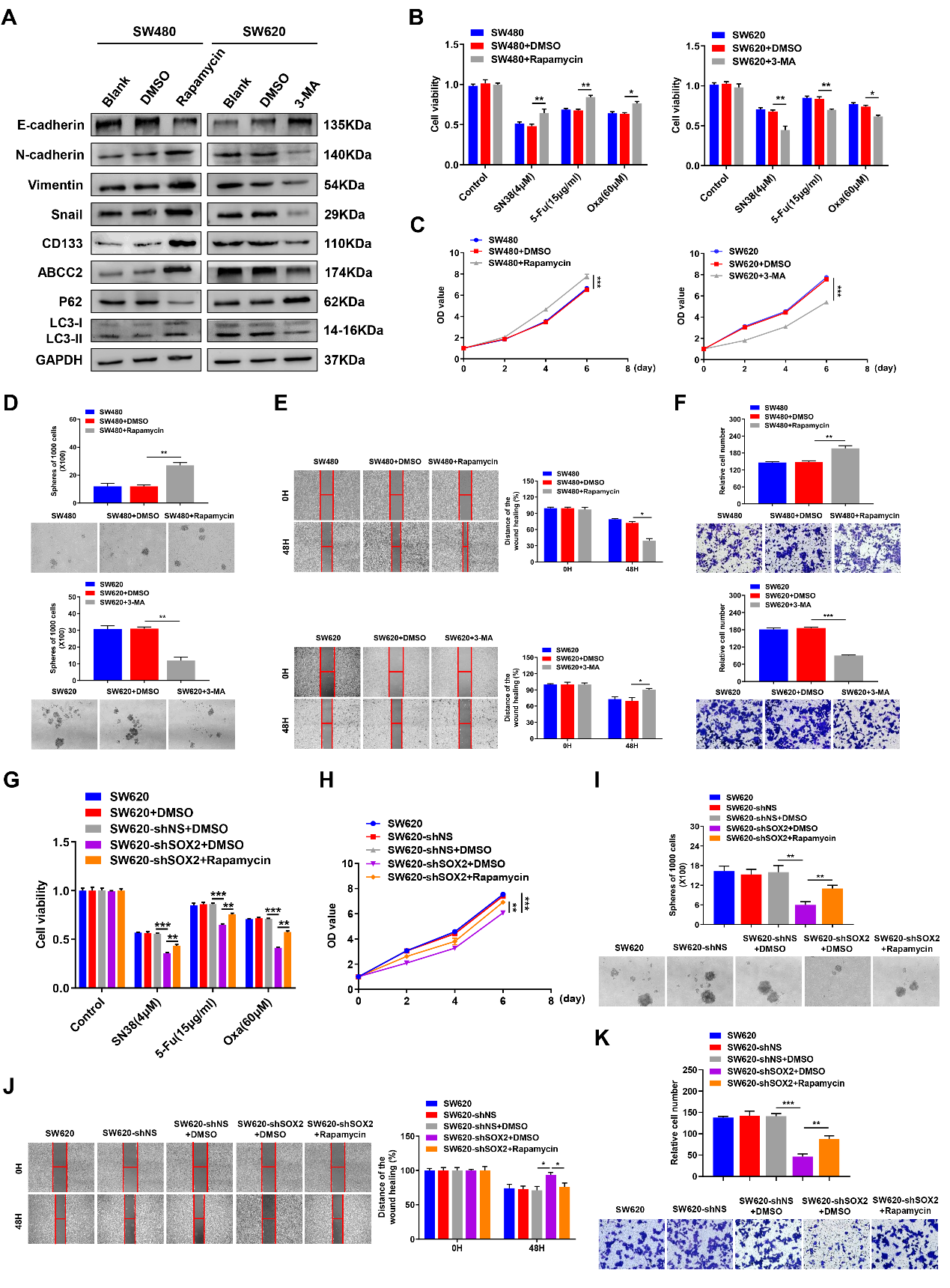


**Supplementary Figure S4. SOX2 promotes chemoresistance, CSCs properties and EMT partly via autophagy in CRC.**

**A,** Activating or inhibiting autophagy with Rapamycin (50nM) or 3-MA (10mM) treatment for 24h was performed in SW480 or SW620 cells, then western blot was conducted with indicated antibodies. **B-F,** The ability of chemoresistance (B), proliferation (C), stemness (D) migration (E) and invasion (F) was examined in SW480 or SW620 cells with autophagy activation or blockage by drug sensitivity assay, cell viability assay, tumor sphere formation assay, wound healing assay and transwell invasion assay, respectively. **G-K,** Activating autophagy was performed in SW620 cells with stable SOX2 deletion by Rapamycin (50nM) treatment for 24h, then the ability of chemoresistance (G), proliferation (H), stemness (I) migration (J) and invasion (K) was examined in SW620 cells with autophagy activation by drug sensitivity assay, cell viability assay, tumor sphere formation assay, wound healing assay and transwell invasion assay, respectively. Experiments were conducted in triplicate. Data are shown as mean ± SEM. *P<0.05, **P<0.01, ***P<0.001.


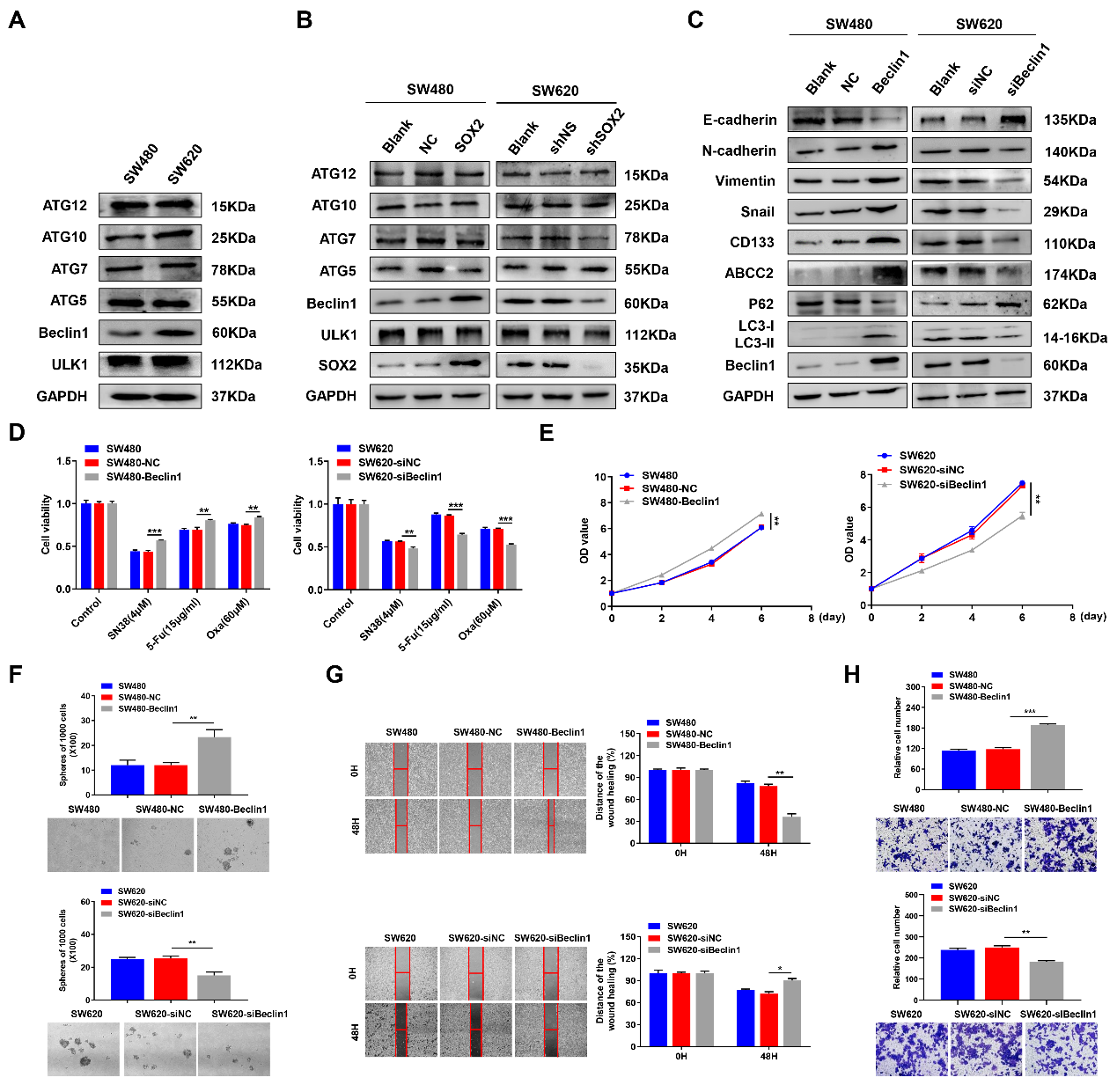


**Supplementary Figure S5.** **Beclin1 promotes autophagy, chemoresistance, CSCs properties and EMT in CRC.**

**A and B,** Western blot analysis of autophagy-related genes expression in SW480 and SW620 cells (A) as well as in SW480 and SW620 cells transfected with SOX2 clone or SOX2 shRNA (B). **C,** Overexpressing or silencing Beclin1 was performed in SW480 or SW620 cells, then western blot was performed with indicated antibodies. **D-H,** The ability of chemoresistance (D), proliferation (E), stemness (F) migration (G) and invasion (H) was examined in SW480 or SW620 cells transfected with Beclin1 clone or Beclin1 siRNA by drug sensitivity assay, cell viability assay, tumor sphere formation assay, wound healing assay and transwell invasion assay, respectively. Experiments were conducted in triplicate. Data are shown as mean ± SEM. *P<0.05, **P<0.01, ***P<0.001.
