# Supplementary material for: SOX2 promotes chemoresistance, cancer stem cells properties, and epithelial–mesenchymal transition by β-catenin and Beclin1/autophagy signaling in colorectal cancer
Source: Cell Death Dis. 2021 May 5;12(5):449. doi: 10.1038/s41419-021-03733-5 (PMC8100126; doi:10.1038/s41419-021-03733-5)
Supplement: Supplementary file 2 — Supplementary Tables [file 41419_2021_3733_MOESM2_ESM.docx]

**Supplementary Tables**

**Supplementary Table S1. The sequences of short hairpin RNA (shRNA).**

| **shRNA** | **Forward** | **Reverse** |
| --- | --- | --- |
| SOX2 RNAi-1 | CCGGCAGCTCGCAGACCTACATGAACTCGAGTTCATGTAGGTCTGCGAGCTGTTTTTG | AATTCAAAAACAGCTCGCAGACCTACATGAACTCGAGTTCATGTAGGTCTGCGAGCTG |
| SOX2  RNAi-2 | CCGGGAAGAAGGATAAGTACACGCTCTCGAGAGCGTGTACTTATCCTTCTTC TTTTTG | AATTCAAAAAGAAGAAGGATAAGTACACGCTCTCGAGAGCGTGTACTTATCCTTCTTC |
| ABCC2  RNAi-1 | CCCCATAGCTTCATTCCTGAGTACTCGAGTACTCAGGAATGAAGCTATGGTTTTTG | AATTCAAAAACCATAGCTTCATTCCTGAGTACTCGAGTACTCAGGAATGAAGCTATGG |
| ABCC2  RNAi-2 | CCGGGCATCTGAAGTCCCTGAGAAACTCGAGTTTCTCAGGGACTTCAGATGCTTTTTG | AATTCAAAAAGCATCTGAAGTCCCTGAGAAACTCGAGTTTCTCAGGGACTTCAGATGC |

**Supplementary Table S2. The sequences of small interfering RNA (siRNA).**

| **siRNA** | **Forward** | **Reverse** |
| --- | --- | --- |
| β-catenin siRNA-1 | GCAGUUGUAAACUUGAUUATT | UAAUCAAGUUUACAACUGCTT |
| β-catenin siRNA-2 | GGACACAGCAGCAAUUUGUTT | ACAAAUUGCUGCUGUGUCCTT |
| Beclin1 siRNA-1 | GGUCUAAGACGUCCAACAATT | UUGUUGGACGUCUUAGACCCT |
| Beclin1 siRNA-2 | GCUCAGUAUCAGAGAGAAUTT | AUUCUCUCUGAUACUGAGCTT |

**Supplementary Table S3. Primer Sequences Used for qRT-PCR.**

| Primers names | Primers sequences (5’→3’) |
| --- | --- |
| Human-GAPDH-Forward | CATCACTGCCACCCAGAAGACTG |
| Human-GAPDH-Reverse | ATGCCAGTGAGCTTCCCGTTCAG |
| Human-SOX2-Forward | CATCACCCACAGCAAATGAC |
| Human-SOX2-Reverse | TTTTTCGTCGCTTGGAGACT |
| Human-ABCA2-Forward | TGTTCTTTATCCTGCTGGGG |
| Human-ABCA2-Reverse | GATTGCATGACAGGCAGGA |
| Human-ABCA3-Forward | CGAGGACTACATTCGCTATGAC |
| Human-ABCA3-Reverse | TGTAACTGAAGCGCAGGTG |
| Human-ABCB1-Forward | AGGCCAACATACATGCCTTC |
| Human-ABCB1-Reverse | CCACCAGAGAGCTGAGTTCC |
| Human-ABCB4-Forward | AGGCAGCGAGGAAACGGAA |
| Human-ABCB4-Reverse | TGCTGATGCTGCCTAGTTCAA |
| Human-ABCB5-Forward | GCGAGCAAAGGTCGGACTACAATCGTGG |
| Human-ABCB5-Reverse | CCCAGAACCACAAAAGGCCATTCAGGC |
| Human-ABCB11-Forward | GCATCGTVTCCCAGGAGCC |
| Human-ABCB11-Reverse | CVGASGTGGCCTCGTCC |
| Human-ABCC1-Forward | GGTCAGCCCAACTCTCTTGG |
| Human-ABCC1-Reverse | CACTAGGGCTACCAGCCAGA |
| Human-ABCC2-Forward | TGCACAAGCAACTGCTGAAC |
| Human-ABCC2-Reverse | AGGCAGGGTGTCATCCACT |
| Human-ABCC3-Forward | TGGAGGAGAAGGACCTCTGG |
| Human-ABCC3-Reverse | GTGCTGCTGAAGCCTTGTG |
| Human-ABCC4-Forward | GAAATTGGACTTCACGATTTAAGG |
| Human-ABCC4-Reverse | TTCCACAGTTCCTCATCCGT |
| Human-ABCC5-Forward | ACGCAGAGATGAGGTACCGA |
| Human-ABCC5-Reverse | ATCCCCAGCGAGGACTTC |
| Human-ABCC6-Forward | CTCCCATCCATCCTTCT |
| Human-ABCC6-Reverse | CCTCGCTACCATACAATATGA |
| Human-ABCC10-Forward | GTCCAGATTACATCCTACCCTGC |
| Human-ABCC10-Reverse | GCCAACACCTCTAGCCCTATG |
| Human-ABCC11-Forward | CTGAAAATGACTAGGAAGAGGACA |
| Human-ABCC11-Reverse | TTATCTCAGTGAAGAAGTGGCTGT |
| Human-ABCG2-Forward | GGTGCCATTTACTTTGGGC |
| Human-ABCG2-Reverse | ACAAAGTTCCACGGCTGA |
| Human-β-catenin-Forward | AAAGCGGCTGTTAGTCACTGG |
| Human-β-catenin-Reverse | CGAGTCATTGCATACTGTCCAT |
| Human-c-Myc-Forward | GGCTCCTGGCAAAAGGTCA |
| Human-c-Myc-Reverse | CTGCGTAGTTGTGCTGATGT |
| Human-cyclin D1-Forward | GCTGCGAAGTGGAAACCATC |
| Human-cyclin D1-Reverse | CCTCCTTCTGCACACATTTGAA |
| Human-Axin2-Forward | CAACACCAGGCGGAACGAA |
| Human-Axin2-Reverse | GCCCAATAAGGAGTGTAAGGACT |
| Human-Beclin1-Forward | CCATGCAGGTGAGCTTCGT |
| Human-Beclin1-Reverse | GAATCTGCGAGAGACACCATC |

**Supplementary Table S4. Primer Sequences Used for** **Chromatin immunoprecipitation.**

| Primers names | Primers sequences (5’→3’) |
| --- | --- |
| ABCC2 promoter-Forward | TGATGCCACCACTCTG |
| ABCC2 promoter-Reverse | GCCAAATGGGAATGAA |
| Beclin1 promoter-Forward | GGGGAAATAGTAGAA |
| Beclin1 promoter-Reverse | GTGACAGAGCGAGAC |

**Supplementary Table S5. Correlation between ABCC2 expression and clinicopathological parameters in CRC patients.**

| **Variables** | **ABCC2 expression** | | **P-value** |
| --- | --- | --- | --- |
|  | **Score<3**  **Low(n=36)** | **Score≥3**  **High(n=54)** |  |
| **Age(years)** |  |  |  |
| <65 | 10 | 24 | 0.110 |
| ≥65 | 26 | 30 |  |
| **Gender** |  |  |  |
| male | 19 | 27 | 0.796 |
| female | 17 | 27 |  |
| **Pathological grade** |  |  |  |
| well-moderate | 33 | 46 | 0.554 |
| poor | 3 | 8 |  |
| **Lymph metastasis** |  |  |  |
| no | 28 | 29 | **0.020** |
| yes | 8 | 25 |  |
| **Distant metastasis** |  |  |  |
| no | 36 | 53 | 0.600 |
| yes | 0 | 1 |  |
| **AJCC stage** |  |  |  |
| Ⅰ/Ⅱ | 28 | 29 | **0.020** |
| Ⅲ/Ⅳ | 8 | 25 |  |
| **Tumor size** |  |  |  |
| <5cm | 15 | 21 | 0.792 |
| ≥5cm | 21 | 33 |  |
| **Tumor number** |  |  |  |
| single | 34 | 50 | 0.544 |
| multiple | 2 | 4 |  |

**Supplementary Table S6. Correlation between Beclin1 expression and clinicopathological parameters in CRC patients.**

| **Variables** | **Beclin1 expression** | | **P-value** |
| --- | --- | --- | --- |
|  | **Score<3**  **Low(n=33)** | **Score≥3**  **High(n=57)** |  |
| **Age(years)** |  |  |  |
| <65 | 11 | 19 | 0.989 |
| ≥65 | 22 | 38 |  |
| **Gender** |  |  |  |
| male | 16 | 30 | 0.705 |
| female | 17 | 27 |  |
| **Pathological grade** |  |  |  |
| well-moderate | 30 | 44 | 0.101 |
| poor | 3 | 13 |  |
| **Lymph metastasis** |  |  |  |
| no | 23 | 31 | 0.153 |
| yes | 10 | 26 |  |
| **Distant metastasis** |  |  |  |
| no | 32 | 58 | 0.184 |
| yes | 1 | 0 |  |
| **AJCC stage** |  |  |  |
| Ⅰ/Ⅱ | 23 | 26 | **0.027** |
| Ⅲ/Ⅳ | 10 | 31 |  |
| **Tumor size** |  |  |  |
| <5cm | 15 | 25 | 0.883 |
| ≥5cm | 18 | 32 |  |
| **Tumor number** |  |  |  |
| single | 33 | 52 | 0.08 |
| multiple | 0 | 5 |  |

**Supplementary Table S7. The chemotherapy effect of irinotecan combined with CQ treatment on tumor in SW620 cells in vivo.**

| **Cell Type** | **SW620 WT** | **SW620 SOX2 KD** |
| --- | --- | --- |
| **Group** | **irinotecan + CQ** | **irinotecan + CQ** |
| **Average tumor volume**  **on the 22^nd^ day (mm^3^)** | **610.99** | **288.01** |
| **Average tumor volume**  **on the 37^th^ day (mm^3^)** | **173.91** | **22.15** |
| **Fold change (37^th^/22^nd^)** | **0.28** | **0.08** |

**Note: 37th/22nd, the average tumor volume on the 37th day versus the average tumor volume on the 22nd day.**
